# Integrated Immunopeptidomics and Proteomics Study of SARS-CoV-2–Infected Calu-3 Cells Reveals Dynamic Changes in Allele-specific HLA Abundance and Antigen Presentation

**DOI:** 10.1016/j.mcpro.2023.100645

**Published:** 2023-09-13

**Authors:** Rui Chen, Kelly M. Fulton, Anh Tran, Diana Duque, Kevin Kovalchik, Etienne Caron, Susan M. Twine, Jianjun Li

**Affiliations:** 1 Human Health Therapeutics Research Centre, National Research Council Canada, Ottawa, Ontario, Canada; 2 CHU Sainte-Justine Research Center, Montreal, Quebec, Canada; 3 Department of Pathology and Cellular Biology, Faculty of Medicine, Université de Montréal, Quebec, Canada

## Abstract

We present an integrated immunopeptidomics and proteomics study of severe acute respiratory syndrome coronavirus 2 (SARS-CoV-2) infection to comprehensively decipher the changes in host cells in response to viral infection. Immunopeptidomics analysis identified viral antigens presented by host cells through both class I and class II MHC system for recognition by the adaptive immune system. The host proteome changes were characterized by quantitative proteomics and glycoproteomics and from these data, the activation of toll-like receptor 3–interferon pathway was identified. Glycosylation analysis of human leukocyte antigen (HLA) proteins from the elution and flow-through of immunoprecipitation revealed that SARS-CoV-2 infection changed the glycosylation pattern of certain HLA alleles with different HLA alleles, showing distinct dynamic changes in relative abundance. The difference in the glycosylation and abundance of HLA alleles changed the number of strong binding antigens each allele presented, suggesting the impact of SARS-CoV-2 infection on antigen presentation is allele-specific. These results could be further exploited to explain the imbalanced response from innate and adaptive immune system in coronavirus disease 2019 cases, which would be helpful for the development of therapeutics and vaccine for coronavirus disease 2019 and preparation for future pandemic.

## Highlights

• Host response to SARS-CoV-2 infection comprehensively deciphered.
• Both class I and class II viral epitopes were identified by immunopeptidomics.
• Activation of TLR3-IFN pathway and downregulation of ACE2 identified.
• Allele-specific changes in HLA identified and correlates with antigen presentation.

## Graphical Abstract

**Figure:**
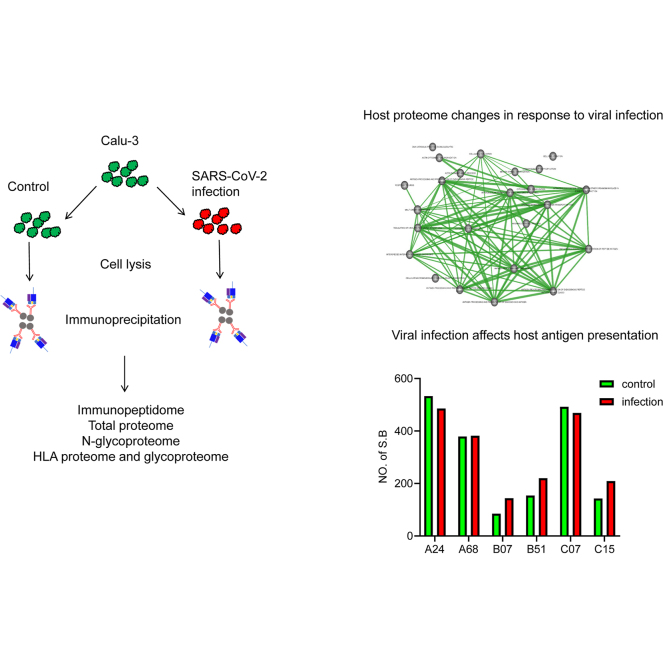

The novel severe acute respiratory syndrome coronavirus 2 (SARS-CoV-2) is the causative agent of coronavirus disease 2019 (COVID-19), which was declared a pandemic in March 2020. Since then, the world has witnessed the mobilization of the global scientific community toward the fastest vaccine development in history. Currently, there are 14 vaccines approved by WHO for emergency use globally, with another 341 in various stages of development. Three years after the discovery of the novel coronavirus, there is still a need to understand how the virus mobilizes the host cellular pathways to allow rapid infection and replication and identify the key viral epitopes that stimulate the host immune system to generate protective immunity.

SARS-CoV-2 is a beta coronavirus, a member of the Coronaviridae family of viruses, which include four subgroups, denoted alpha, beta, gamma, and delta. Together, the family infect a wide range of mammal and bird species, with mutations allowing the viruses to cross the species barrier. SARS-CoV-2 has a 30 kb positive–sense RNA genome, with 14 ORFs that encode a total of 29 proteins. These include four structural proteins (spike, envelope, membrane, and nucleocapsid protein), 16 nonstructural proteins (Nsp 1–16) and nine accessory proteins (Orf 3a, Orf 3b, Orf6, Orf 7a, Orf 7b, Orf8, Orf 9b, Orf 9c, and Orf 10) (1). Built on the knowledge of related coronaviruses, recent studies have illuminated the life cycle of SARS-CoV-2 (2). This virus enters cells *via* the binding of the spike structural protein to human angiotensin–converting enzyme 2 (ACE2) on the surface of host cells. Intracellularly, the viral genome is transcribed by the host ribosomes. The viral genome is replicated by the viral RNA–dependent RNA polymerase, on membrane structures derived from the endoplasmic reticulum (ER). Assembly of mature virions occurs in the ER-Golgi intermediate compartment, budding from the ER-Golgi membranes before been released outside the cells *via* an exocytosis like process (3). Despite the rapid progress made by the scientific community, the emergence of variants of concern (VoCs) continues to challenge vaccine and drug developers. Although the currently approved vaccines showed excellent efficacy toward the original SARS-CoV-2, vaccine breakthrough infections have been observed with several VoCs. This continues to highlight the need to build on our current knowledge of the host response to the virus, in particular the modified signaling pathways to allow viral infection and viral epitopes presented to the immune system.

The genome sequence of SARS-CoV-2 was widely disseminated in the early 2020 (4), supporting the rapid design of first-generation vaccines. Infection with the novel coronavirus elicits both humoral and cell-mediated immunities. Although vaccines offer protection against the original virus and some protection in terms of reduced severity of infection and transmission of VoC, there is still a need for booster vaccines and universal vaccines that offer protection from current and emerging variants of concern. There are also concerns about viral resistance to antibody defenses. There remains an ongoing need to understand immune responses to viral infection, for example, how cytotoxic T cells could boost immunity against new variants (5). Viruses infect host cells and their proteins are processed and presented on the surface of human cells by major histocompatibility complex (MHC). Cytotoxic T cells recognize nonself antigens and initiate an immune response that clears the infected cells. The totality of the peptides presented on MHC is known as the immunopeptidome and improving knowledge of the immunopeptidome will shed light on those peptides that activate cytotoxic T cells. This will require the identification of the viral peptide epitopes presented to the host immune system. Advanced *in silico* methods are used to predict immune epitopes, but it is not known whether these epitopes confer protective immunity or stimulate T cell response. Additionally, *in silico* methods cannot yet take into account the ways that viruses can manipulate the processing and presentation of viral antigens by host cells. For example, viruses have been shown to downregulate the proteasome, host cell protein translation, and human leukocyte antigen I (HLA-I) expression (6). In addition, prediction modeling does not take into account kinetics of viral suppression on HLA-I expression, which may mean that there is a larger repertoire of viral epitopes early on during infection of host cells (7). As a result, studies typically use overlapping peptide tiling and/or *in silico* prediction approaches to forecast HLA-I binding peptides (8, 9, 10, 11, 12, 13).

While *in silico* prediction provides a richness of information regarding immune epitopes, the information needs to be confirmed experimentally and the identified peptides need to be tested for their ability to activate T cells. For this purpose, mass spectrometric sequencing of peptides is required. Mass spectrometry (MS) can be used to identify viral peptides bound to MHCs and it is a direct and targeted method to discover endogenously presented peptides. The approach has been used in other viral infections and revealed new antigens that activated T cell responses against West Nile virus (14), HIV (15, 16), and measles (17). It was reported that epitopes of SARS-CoV-2 derived not only from structural but also nonstructural genes in regions which are highly conserved among SARS-CoV-2 strains, including recently recognized variants (18). This included epitopes from membrane glycoprotein and nonstructure protein 13 (NSP13). A recent study investigated the HLA-I immunopeptidome of two SARS-CoV-2–infected human cells lines and identified HLA-I–bound peptides derived from canonical and noncanonical ORFs (19), which are not reflected in current vaccines. Other studies have combined HLA peptide sequencing with host cell proteomics, which showed that those viral proteins expressed early in infection were found more frequently in HLA presentation (20).

In addition to immunopeptidomics, proteomics analysis, which studies the changes of host proteome after infection, can provide more insights into the molecular basics of SARS-CoV-2 infection. Several reports used different cell lines to study the change in either global proteome or post-translational modfication -specific subproteome to identify the pathways involved in response to viral infection. A deeper understanding of the activated pathways can be used to develop therapeutics against COVID-19 (21, 22, 23, 24, 25). However, immunopeptidomics were not included in those studies. To date, most immunopeptidomics studies focused on the analysis of MHC-associated peptides (19, 26, 27). As shown in recent studies, SARS-CoV-2 infection can hijack the antigen presentation pathways and prevent infected cells from presenting viral antigens (28, 29), which might cause the impaired T cell response in COVID-19 patients (30). Thus, it is important to study how the infection impacts the antigen presentation, including the changes in HLA proteins, which is vital to the adaptive immune system. To fill this gap, we developed an integrated immunopeptidomics and proteomics approach that uses MHC immunoprecipitation and MS to survey the SARS-CoV-2 immunopeptidome and changes in the host proteome. In additional to the peptide-centric immunopeptidomics, the HLA proteins from immunoprecipitation were also analyzed by LC-MS/MS. Combined with glycosylation analysis of HLA proteins from both the elution and flow-through from MHC immunoprecipitation, significant difference in glycosylation from HLA alleles was found between control and infected cells. The difference in the glycosylation pattern and abundance might lead to changes in antigen presentation as demonstrated by immunopeptidome analysis. This finding suggested that SARS-CoV-2 infection will affect antigen presentation in host cells and this impact was allele-specific.

## Experimental Procedures

### Cell Culture and Infection

Calu-3 cells were cultured in 5%%CO_2_ at 37 °C and in Dulbecco's modified Eagle's medium (DMEM) supplemented with 10% fetal bovine serum, 50 IU/ml penicillin, 50 μg/ml streptomycin, and 2 mM L-glutamine. Cells were allowed to grow to 80 to 90% confluence in T175 flasks. All the cells used in this study were from one batch and split into six aliquots of which three were infected by SARS-CoV-2. Vero and Vero E6 cells were cultured in DMEM supplemented with 1× nonessential amino acid, 20 IU/ml penicillin, 0.02 mg/ml streptomycin, 1 mM sodium pyruvate, and 5% fetal bovine serum. SARS-CoV-2 isolate Canada/ON/VIDO-01/2020 was propagated on Vero E6 cells and titered on Vero cells. Exact genetic identity to original isolate was confirmed by whole viral genome sequencing. Passage three virus stocks were used in all subsequent experiment that required live virus. For cell infection, cells were washed with PBS and infected by SARS-CoV-2 at a multiplicity of infection of 5 at 37 °C for 1 h in infection media (DMEM supplemented with 1× nonessential amino acid, 20 IU/ml penicillin, 0.02 mg/ml streptomycin, 1 mM sodium pyruvate). After infection, cells were washed with infection media and then cultured in 30 ml infection media for a further 24 h at 37 °C. For controls, only media were changed and cells were kept for 24 h.

### Immunoprecipitation

Both control and infected Calu-3 cells were washed with 10 ml PBS three times to remove media. Five microliters of lysis buffer (20 mM Tris–HCl, pH = 7.4, 100 mM NaCl, 5 mM MgCl_2_, 0.5% CHAPS, 1.5% Triton X-100) with protease and phosphatase inhibitor was added to each flask. Cells were lysed by shaking the flask. Detached cells were transferred into 50 ml tube and rotated for 1 h at 4 °C. Cell lysate was cleared by centrifugation at 16,000*g* for 10 min, and supernatant was transferred into a new tube for immunoprecipitation.

W6/32 (MHC I) and L243 (MHC II) antibodies were conjugated to AminoLink plus agarose beads following the manufacture’s instruction and 1 ml of resins was packed into a cartridge for immunoprecipitation. Cell lysates were firstly passed through an MHC I affinity column and the flow-through was immediately loaded onto MHC II affinity column. Flow-through from each immunoprecipitation was collected for proteomics analysis. Both MHC I and MHC II affinity columns were washed with 10 column volumes of wash buffers as described previously (31) and MHC-peptides complex was eluted with 5 ml of 10% acetic acid. MHC-associated peptides were purified with C18 cartridge and eluted with 30% acetonitrile (ACN). Eluted peptides were lyophilized by Speed Vac and reconstituted in 0.1% formic acid for LC-MS/MS analysis. The remaining protein fraction bound to column from immunoprecipitation was further eluted from the C18 cartridge by 80% ACN and lyophilized by Speed Vac. Dried protein fraction was reconstituted with 8 M urea in 100 mM Tris–HCl (pH = 7.4), reduced by dithiothreitol, alkylated by iodoacetamide, and digested by trypsin. Protein digest was desalted by HyperSep C18 solid-phase extraction (SPE) cartridge, and eluted protein digest was dried by Speed Vac for further process.

### Sample Preparation for Proteomics Analysis

Cell lysate from the flow-through of immunoprecipitation was collected for proteomics analysis. Proteins from the flow-though were precipitated by adding 10 volumes of cold acetone and incubated overnight at −20 °C. Pelleted protein precipitate was washed with acetone for three times and air dried in a fume hood. For digestion, proteins were solubilized with 8 M urea in 100 mM Tris–HCl (pH = 7.4) and protein concentration was measured by DC Assay. Proteins were reduced by dithiothreitol, alkylated by iodoacetamide, and digested by trypsin at a ratio of 25:1 (protein to enzyme). Protein digest was desalted by HyperSep C18 SPE cartridge, and eluted protein digest was dried by Speed Vac for further process.

Hydrophilic interaction SPE(HILIC SPE) was conducted as previously described with minor changes (32). Two hundred micrograms of dried protein digest was solubilized in 80% of ACN with 1% of trifluoroacetic acid and loaded onto the microcolumn packed with 5 mg of polyhydroxyethyl beads (100 Å, 5 μm). After three washes with the same loading buffer, glycopeptides retained on SPE column were eluted by 30% of ACN with 0.1% trifluoroacetic acid. For the flow-through lysate digestion, two replicates of HILIC enrichment were carried out, and eluted glycopeptides from one replicate were lyophilized for intact glycopeptides analysis. Glycopeptides from the other replicate were then deglycosylated by incubation with 50 μl of 50 mM Tris–HCl (pH = 7.5) with 1 unit per microliter of PNGase F (New England Biolabs) overnight at 37 °C. Deglycosylated peptides were dried with Speed Vac, and the samples were stored at −80 ^°^C until MS analysis. For HLA protein from immunoprecipitation, flow-through and wash from HILIC SPE were combined to collect nonglycosylated peptides for protein identification. Elution from HILIC SPE was lyophilized for intact glycopeptides analysis.

### LC-MS/MS Analysis

All the samples were analyzed by an LC-MS/MS system comprised of an Exploris 480 mass spectrometer (Thermo Fisher Scientific Inc) and an Ultimate 3000 ultra-high-performance liquid chromatography. Peptides were loaded *via* an Acclaim PepMap 100 trap column (C18, 5 μm, 100 Å, Thermo Fisher Scientific) onto a Waters BEH C18 column (100 μm × 100 mm) packed with reverse phase beads (1.7 μm, 120 Å pore size, Waters). Different gradients of 15, 45, or 90 min from 5% to 30% ACN (v/v) containing 0.1% formic acid (v/v) was performed at an eluent flow rate of 500 nl/min to analyze HLA protein digest, immunopeptidome, and total proteome, respectively. For data-dependent acquisition, a full ms1 scan (resolution: 60, 000; automatic gain control target: standard; maximum injection time: 50 ms; scan range: 375–1600 *m/z*) was preceded by subsequent ms2 scans (resolution: 15,000; automatic gain control target: standard; maximum injection time: 150 ms; isolation window: 2 *m/z*; scan range: 200–2000 *m/z*; normalized collision energy: 30). To minimize repeated sequencing of the same peptide, the dynamic exclusion was set to 60 s, and the “exclude isotopes” option was activated.

### Database Search and Analysis

For immunopeptidomics, database search was performed by MSfragger 3. 0 (33) against UniProt human protein database (downloaded on September 9th, 2021 with 20,199 entries) with common Repository of Adventitious Proteins, plus the protein sequences from SARS-CoV-2 (downloaded from UniProt on September 9th, 2021 with 16 entries). Both precursor and fragment tolerances were set at 20 ppm. Methionine oxidation was set as variable modification. Nonspecific cleavage was set and peptides with 7 to 25 amino acids were retained. All peptide-spectrum matches (PSMs) were filtered with a 5% false discovery rate (FDR), and the identified peptides were further filtered by peptide length (8–14 for MHC I peptides and 12–25 for MHC II peptides). Peptides from HLA, beta-2 microglobulin, and contaminant proteins were also removed. Binding of 9mers to HLA alleles was predicted by NetMHCpan 4.0 (34).

Data from proteomics analysis of HLA proteins that collected from MHC immunoprecipitation was firstly searched by MSfragger 3.0 using the same protein database described above. Trypsin cleavage at KR/P was set for protease specificity. Both precursor and fragments tolerance were set at 20 ppm. Carbamidomethylation of cysteine was set as fixed modification, and methionine oxidation was set as variable modification. For the comparison of the relative abundance of each allele, a customized protein database was generated by replacing the sequences of all HLA-A, HLA-B, and HLA-C proteins from the UniProt protein database with A∗68:01, A∗24:02, B∗07:02, B∗51:01, C∗07:02, and C15∗:02 sequences from IPD IMGT/HLA database (35) (release 3.41.2, https://www.ebi.ac.uk/ipd/imgt/hla/). The customized database was further used for the database search of N-glycoproteome and total proteome from the flow-through of immunoprecipitation for the relative quantification of each HLA allele. For the analysis of HLA proteins from immunoprecipitation, the percentage of each allele among all HLA alleles was calculated by dividing its razor intensity by the combined intensity from all alleles. This percentage was used as normalized abundance of each allele from each experiment.

Data from intact glycopeptides analysis was processed by MSfragger 3.0 using N-glyco-high-energy collision dissociation workflow against the same database described above (36). Trypsin cleavage at KR/P was set for protease specificity. Both precursor and fragment tolerance were set at 20 ppm. Carbamidomethylation of cysteine was set as fixed modification. Methionine oxidation was set as variable modification. For variable modification search of N-glycan data, 182 glycan masses were specified as variable modifications on N-X-S/T (36). Identified glycan compositions were matched to the known glycan structures found in human. One percent FDR was applied to filter PSM, peptide, and protein identifications and all glycan compositions were verified manually. Relative quantification of intact glycopeptides was achieved by extracting the peak area of identified glycopeptides with 10 ppm mass tolerance with Xcalibur (https://www.thermofisher.com/order/catalog/product/OPTON-30965).

For proteomics analysis of the flow-through lysate, RAW data was processed with MaxQuant 1.5.2.8 (https://www.maxquant.org) (37) and searched against the same database used for immunopeptidomics by Andromeda engine (38). Precursor and fragment tolerances were set at 10 ppm and 20 ppm, respectively. For total proteomics, carbamidomethylation of cysteine was set as fixed modification. Methionine oxidation was set as variable modification. For N-glycoproteomics, deamidation of asparagine was set as variable modification. An FDR of 1% was set for PSM, site, and protein identification. Label-free quantification (LFQ) was selected to extract the peak intensities of each N-glycosite, and the results were processed by Perseus. Paired student *t* test was used to compare the abundance of proteins and glycosites. Functional analysis was performed by STRING (https://string-db.org/) (39), and the results were visualized by Enrichment Map (40) within the Cytoscope environment (41). Volcano plots were made by VolcaNoseR (42).

### Experimental Design and Statistical Rationale

All experiments were performed in triplicates for both control and infected Calu-3 cells. Statistical analysis was conducted using paired student *t* test within GraphPad Prism 9 (https://www.graphpad.com/features), and *p* < 0.05 was considered to be statistically significant.

## Results

### Immunopeptidomics-Identified Viral Epitopes Presented on Both MHC I and MHC II

**Fig. 1:**
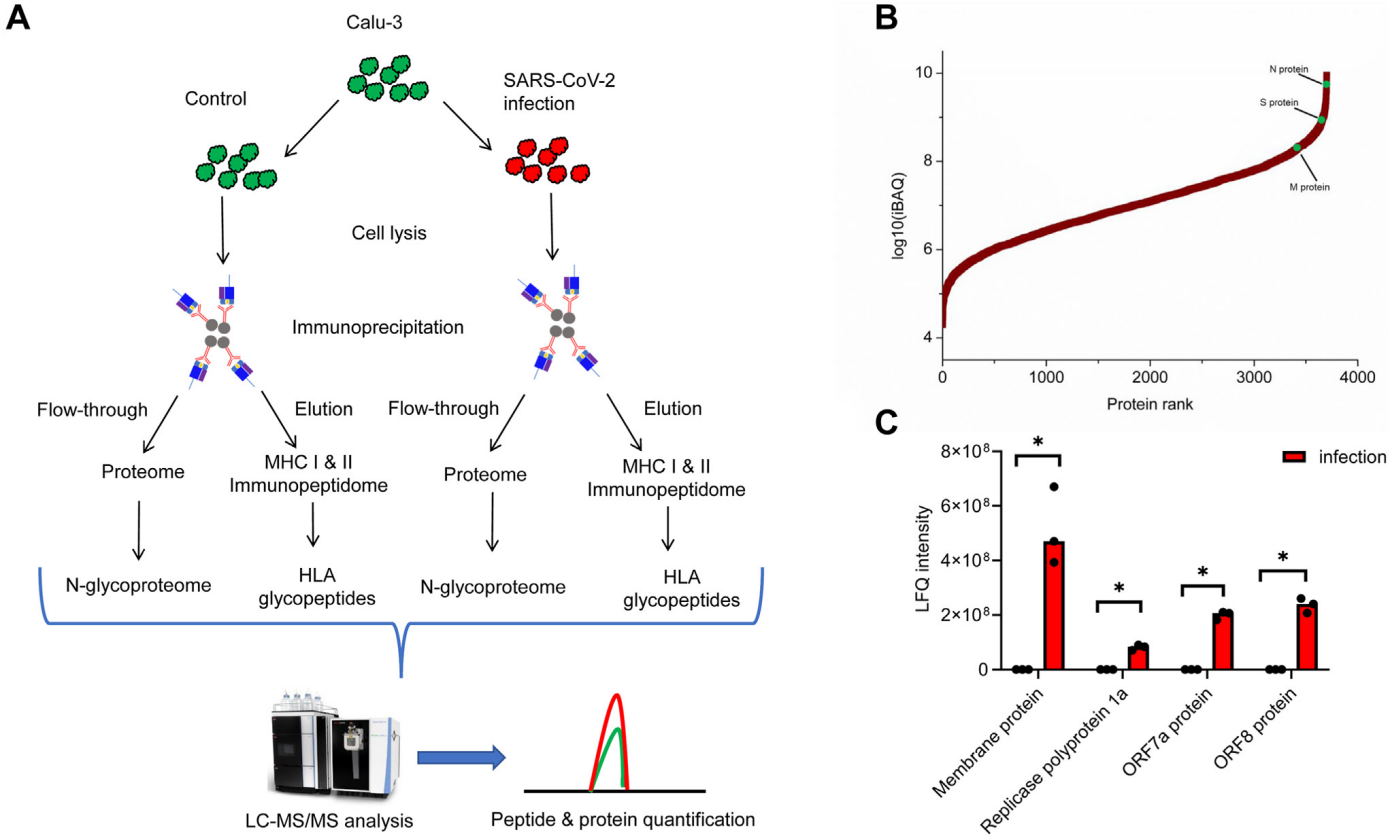
**Integrated immunopeptidomics and proteomics study of SARS-CoV-2 infection.***A*, scheme for the integrated immunopeptidomics and proteomics analysis of SARS-CoV-2 infection of Calu-3 cells. *B*, iBAQ quantification estimates the abundance of viral proteins in infected cells. *C*, abundance of viral proteins from control and infected Calu-3 cells. iBAQ, intensity-based absolute quantification; SARS-CoV-2, severe acute respiratory syndrome coronavirus 2.

We designed an integrated immunopeptidomics and proteomics approach to characterize the interaction between SARS-CoV-2 and host cells. Human lung epithelial cell line Calu-3 was infected by the novel coronavirus at multiplicity of infection of 5 for 24 h. Then both the MHC class I and II immunopeptidome from control and infected cells were captured by immunoprecipitation with W6/32- and L243-conjugated agarose beads, respectively. Eluents from immunoprecipitation were further desalted and fractioned to keep immunopeptides and HLA proteins separately. The flow-through from immunoprecipitation were collected for proteomics analysis (Fig. 1*A*, scheme for the workflow). Purified immunopeptides were analyzed by LC-MS/MS, and immunopeptidome data were searched against a combined protein database from SARS-CoV-2 and human. A total of 2695 and 2699 class I unique peptides were identified from control and infected cells, respectively. For class II peptides, 2632 and 2473 unique peptides were identified from control and infected cells, respectively. The accurate identification of immunopeptides was illustrated by the length distribution of both class I and class II peptides. For MHC I peptides, over 90% of identified peptides were found with 8 to 12 amino acids and the nearly 95% of identified MHC II peptides had 12 to 25 amino acids (Supplemental Fig. S1). The immunopeptidome from the host between control and infected cells showed high level of overlap as similar percentage (60%) of MHC I and MHC II peptides were identified in both control or infected cells (Supplemental Fig. S2), which demonstrated the reproducibility of the proposed immunopeptidomics workflow. Within the immunopeptidome, three class I and two class II peptides from SARS-CoV-2 were identified from infected cells and none of these peptides were identified from control experiments. The identification of viral epitopes was verified by manual interpretation of tandem mass spectrometry (MS/MS) spectra (Supplemental Fig. S3). Peptide TGSNVFQTR from spike glycoprotein (638–646) was predicted to bind to A∗68:01 allele and it locates within the SD1 domain. Peptide ITFGGPSDSTGSNQNGER from nucleocapsid protein (15, 16, 17, 18, 19, 20, 21, 22, 23, 24, 25, 26, 27, 28, 29, 30, 31, 32) was also identified and has been predicted to bind to the same allele (26). Additionally, two class II peptides SPDDQIGYYRRATRRIR and SPDDQIGYYRRATRR from nucleocapsid protein were identified, which has not been reported before.

### SARS-CoV-2 Infection Impacts Antigen Presentation and Activates Interferon Signaling Pathway

**Fig. 2:**
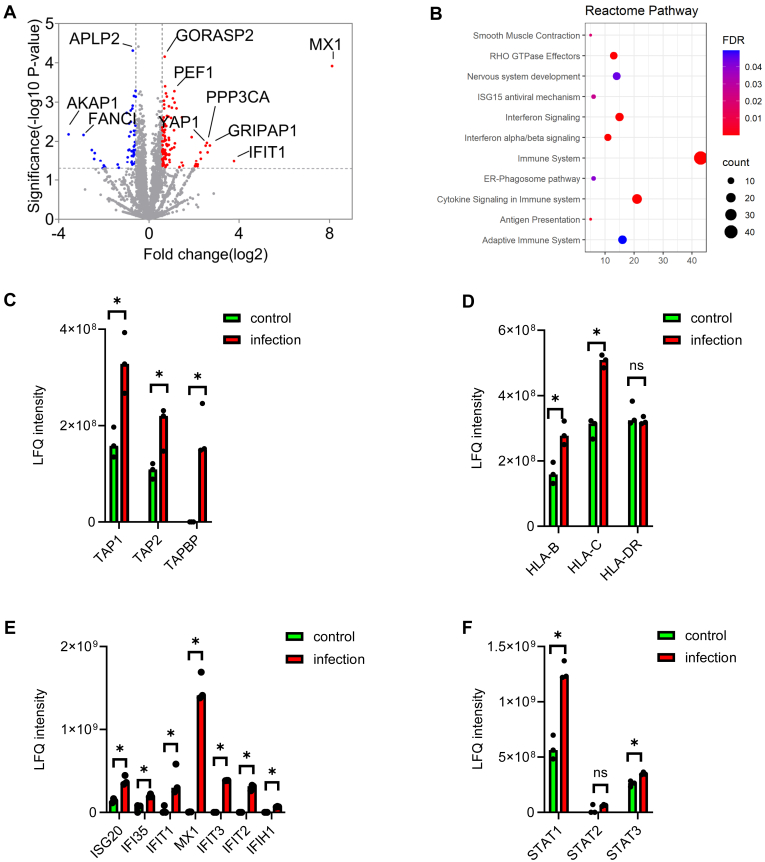
**SARS-CoV-2 infection reshaped the proteome of Calu-3 cells.***A*, *volcano plots* of host proteins shows the changes in proteome. *B*, Reactome pathway analysis reveals activation of type I interferon signaling pathways and immune response to virus infection and secretion of cytokines. *C*, increased abundance of antigen processing proteins in infected cells. *D*, abundance of HLA-B and HLA-C are significantly higher in from the infected cells, while there is no difference in the abundance of HLA-DR. *E*, increased expression of interferon stimulated genes in infected cells. *F*, increased expression of STATs in infected cells. HLA, human leukocyte antigen; SARS-CoV-2, severe acute respiratory syndrome coronavirus 2.

To characterize the host response to viral infection and study the mechanism by which the infection affects the antigen presentation, additional proteomics studies were carried out. Proteins were collected from flow-through of immunoprecipitation by acetone precipitation, followed by in-solution trypsin digestion. Protein digest from each sample was analyzed by LC-MS/MS with data-dependent acquisition and the relative abundance of proteins was quantified by LFQ with Maxquant. We evaluated the reproducibility of the workflow by plotting the LFQ intensities of identified proteins from both control and infection groups (Supplemental Fig. S4). The results showed a high correlation (R^2^ > 0.98, *p* < 0.001 by linear regression) between biological replicates within each group, confirming that immunoprecipitation did not affect the proteomics analysis of the flow-through fraction. With an FDR of 1% at both peptide-spectrum match and protein level, a total of 4479 proteins were identified, including seven proteins from SARS-CoV-2. Among those proteins, ORF9b, nucleocapsid protein, and spike glycoprotein were identified with a LFQ intensity over 10^9^. Intensity-based absolute quantification revealed that the most abundant viral protein, nucleocapsid protein, ranked 18th in all the identified proteins, while spike glycoprotein ranked 481st (Fig. 1*B*). Other viral proteins like membrane protein, replicase polyprotein 1a, ORF7a, and ORF8 protein were found with lower abundance (Fig. 1*C*). Overall, 183 proteins were found with significantly different abundances between control and infected cells by student’s *t* test (fold change > 1.5, *p* < 0.05) (Fig. 2*A*). Reactome pathway analysis identified there were significant enrichment of pathways implicated in both innate and adaptive immune response to infection. These pathways involved in response to virus, including binding to virus and processing of viral proteins for antigen presentation (Fig. 2*B*). Of note, proteins participated in antigen processing (TAP1, TAP2, and TAPBP) were found with doubled expression in infected cells (Fig. 2*C*). Increased levels of HLA-B and HLA-C (1.5 folds, *p* < 0.05) were found in infected cells, whereas no significant change in abundance of HLA-A and HLA-DR was found between control and infected cells (Fig. 2*D*). Since the HLAs were detected from the flow-through of MHC immunoprecipitation, the increased level of HLA-B and HLA-C indicated that more HLA-B and HLA-C did not form MHC–peptide complexes in infected cells compared with HLA-A. Activated interferon (IFN) signaling pathway, which plays an important role of innate immune system and pathway related to cytokine signaling, were also found with Reactome pathway analysis, indicating the secretion of cytokines during infection. The activation of the immune system also triggered expression of interferon-stimulated genes (ISGs) as seven proteins considered as ISG were found with significantly increased expression in infected cells (Fig. 2*E*). Additionally, three members of signal transducer and activator of transcription (STAT) were identified with varied abundance changes in which STAT1 had an over two folds change in infected cells and STAT3 had only subtle change (1.2 folds, *p* < 0.05). Interestingly, no significant difference from STAT2 was detected (Fig. 2*F*).

### Glycoproteomics Suggests Downregulation of ACE2 and Increased Level of TLR3

**Fig. 3:**
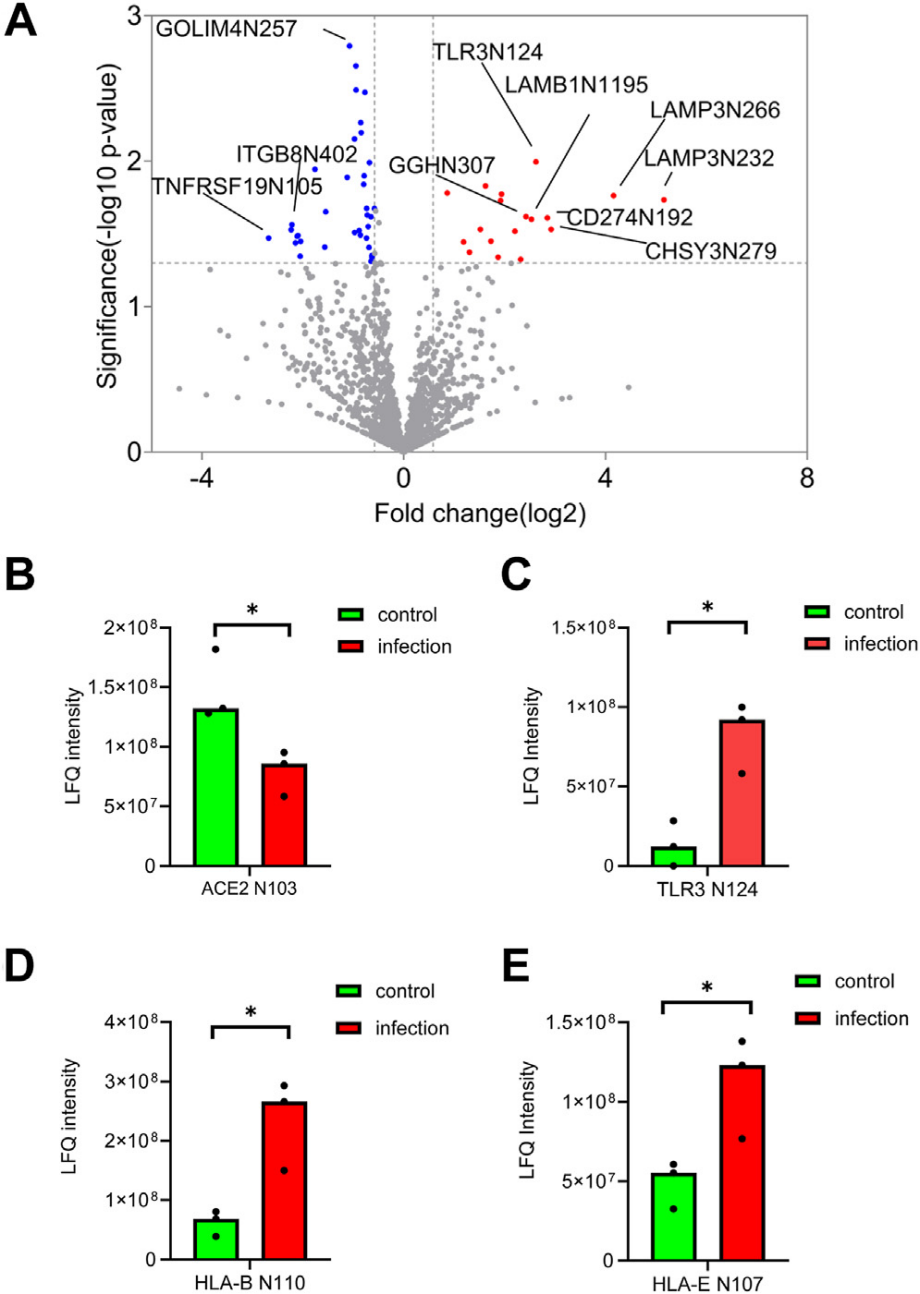
**Glycoproteomics identified changes in the abundance of membrane proteins.***A*, *volcano plots* of N-glycosites quantified by glycoproteomics analysis. statistics of N-glycosites from proteins that involved in immune response to SARS-CoV-2 infection, including ACE2 (*B*), TLR3 (*C*), HLA-B (*D*), and HLA-E (*E*). ACE2, angiotensin-converting enzyme 2; HLA, human leukocyte antigen; SARS-CoV-2, severe acute respiratory syndrome coronavirus 2; TLR, toll-like receptor.

It is well known that SARS-CoV-2 enters host cells through the binding of its spike glycoprotein to ACE2 on the surface of host cells (43). Thus, it is also important to study the changes in the cell surface proteome to identify the receptors that sense the viral infection and trigger downstream signaling. For this purpose, we applied quantitative glycoproteomics approach to analyze the changes in cell surface proteome. In total, 2142 N-glycosites with N#XS/T motif from 876 glycoproteins were identified and the majority of them were annotated as cell surface and membrane proteins by Gene Ontology analysis (Supplemental Fig. S5). However, the statistics demonstrated that there was less change in the surface proteome as only 91 N-glycosites showed significant changes in infected cells compared to control cells (Fig. 3*A*). Further information of those N-glycosites were summarized in Supplemental File S3. Interestingly, the abundance of N-glycosite Asn103 from ACE2 dropped by 2-folds in infected cells compared with control cells (Fig. 3*B*). Other glycoproteins involved in immune response to viral infection were also identified, including toll-like receptor 3 (TLR3). The abundance of its Asn124 N-glycosite increased by 5-folds in infected cells and the other 7 N-glycosites showed consistent trends (Fig. 3*C* and Supplemental Fig. S6). This result suggested that there was increased TLR3 expression in infected cells. Differences in N-glycosite level were also found with HLA proteins. N-glycosite from HLA-B was found with increase abundance in infected cells (Fig. 3*D*). The N-glycosite of nonclassical MHC molecule HLA-E was also found with increased level from the proteome sample in infected cells (Fig. 3*E*), while no significant difference was detected in the N-glycosites of HAL-A, HLA-C and HLA-DR (Supplemental File S3).

### Global and Targeted Glycoproteomics Analysis Reveals Dynamic Changes in Allele-specific HLA Abundance

**Fig. 4:**
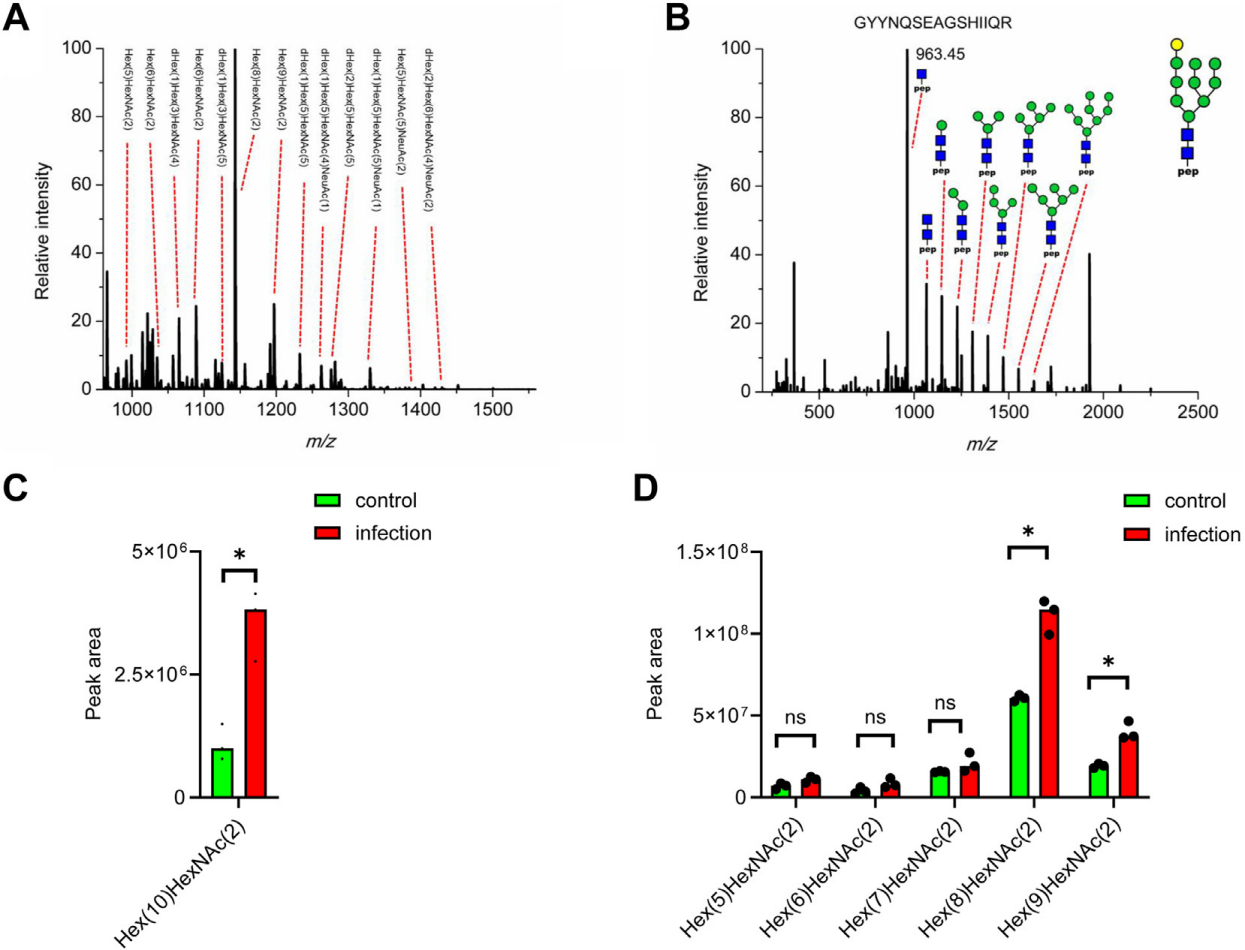
**Glycosylation analysis of HLA glycosylation reveals possible HLA degradation after infection.***A*, glycan compositions identified on HLA C∗:15 glycopeptide (GYYN#QSEAGSHIIQR) captured by immunoprecipitation. *B*, MS/MS spectrum of mono-glucosylated HLA C∗15 glycopeptide in infected Calu-3 cells from the flow-through of MHC immunoprecipitation. *C*, quantification of glycopeptides from the flow-through of MHC immunoprecipitation reveals increased level of mono-glucosylation on C∗:15 glycopeptide after SARS-CoV-2 infection. *D*, statistics of normal high-mannose type glycans from HLAs showed no significantly difference between control and infected cells on C∗:15 glycopeptide from the flow-through of MHC immunoprecipitation. HLA, human leukocyte antigen; MHC, major histocompatibility complex; MS/MS, tandem mass spectrometry; SARS-CoV-2, severe acute respiratory syndrome coronavirus 2.

To further study how did abundance level and glycosylation pattern of MHC-I change after infection, we expanded the glycoproteomics to study the glycosylation of HLA proteins from both immunoprecipitation and the flow-through lysate. Immunoprecipitated proteins represent the HLAs that are bound to peptides within MHC on the cell surface. HLA proteins in the flow-through lysate represent “free” intracellular HLAs not involved in protein–peptide complex formation. In conventional immunopeptidomics workflow, only the peptides that bound to MHC are analyzed, while the HLA proteins are usually discarded. This leads to the loss of useful information on the structure and interaction of HLA proteins. In this study, HLA proteins from MHC immunoprecipitation were collected during C18 desalting, digested by trypsin and analyzed by LC-MS/MS. Database search showed high sequence coverage of HLA proteins (Supplemental Fig. S7) and most HLA alleles were identified with multiple unique peptides (Supplemental File S4). The proteomics analysis of HLA proteins also identified the proteins that interacted with MHCs. Because the proper folding and antigen incorporation of HLA proteins are dependent on the glycosylation (44, 45), the glycosylation of HLA proteins from both the elution and flow-through of immunoprecipitation process were analyzed. Intact glycopeptides were enriched by HILIC-SPE and analyzed by LC-MS/MS. Using MSFraggerGlyco for database search, the glycans attached to HLA proteins were characterized, and the results clearly showed difference in glycosylation from immunoprecipitation and the flow-through lysate that exemplified by glycans found in HLA-C∗15 glycopeptide. HLA proteins from the MHC-peptide elution were mostly modified with hybrid and complex glycans (Fig. 4*A*) (44, 46). In contrast, HLA proteins from the flow-through lysate, theoretically “free” of MHC and peptides, were found with mostly high-mannose type glycans (Supplemental Fig. S8), including the glycoform with mono-glucosylation (Hex (10) HexNAc (2), Fig. 4*B*). Although glycan analysis showed little difference between the immunoprecipitated HLA proteins from control and infected cells (Supplemental Fig. S9). From the flow-through lysate, higher level of mono-glucosylated glycopeptide (fold change ratio of 3.2) was found in infected cells compared with control (Fig. 4*C*). Glycopeptides with normal high-mannose type glycans (Hex (5) HexNAc (2) to Hex (9) HexNAc (2)) only increased by 2-folds (Fig. 4*D*), and the ratio was similar to the fold change from the deglycosylated peptide of HLA-C∗:15 allele, which suggested there was higher level of mono-glucosylation from HLA-C∗:15 after infection. Moreover, glycopeptides with fewer than five hexoses (Supplemental Fig. S10) were only detected in infected cells (Supplemental File S6). In summary, the glycosylation analysis suggested that there was higher level of mono-glucosylation in infected cells and indicated the disruption of MHC formed by HLA-C∗:15 allele, which was consistent with increased level of HLA-C protein from the flow-through of immunoprecipitation.

### Allele-specific Difference in Antigen Presentation Associated with HLA Abundance Change

**Fig. 5:**
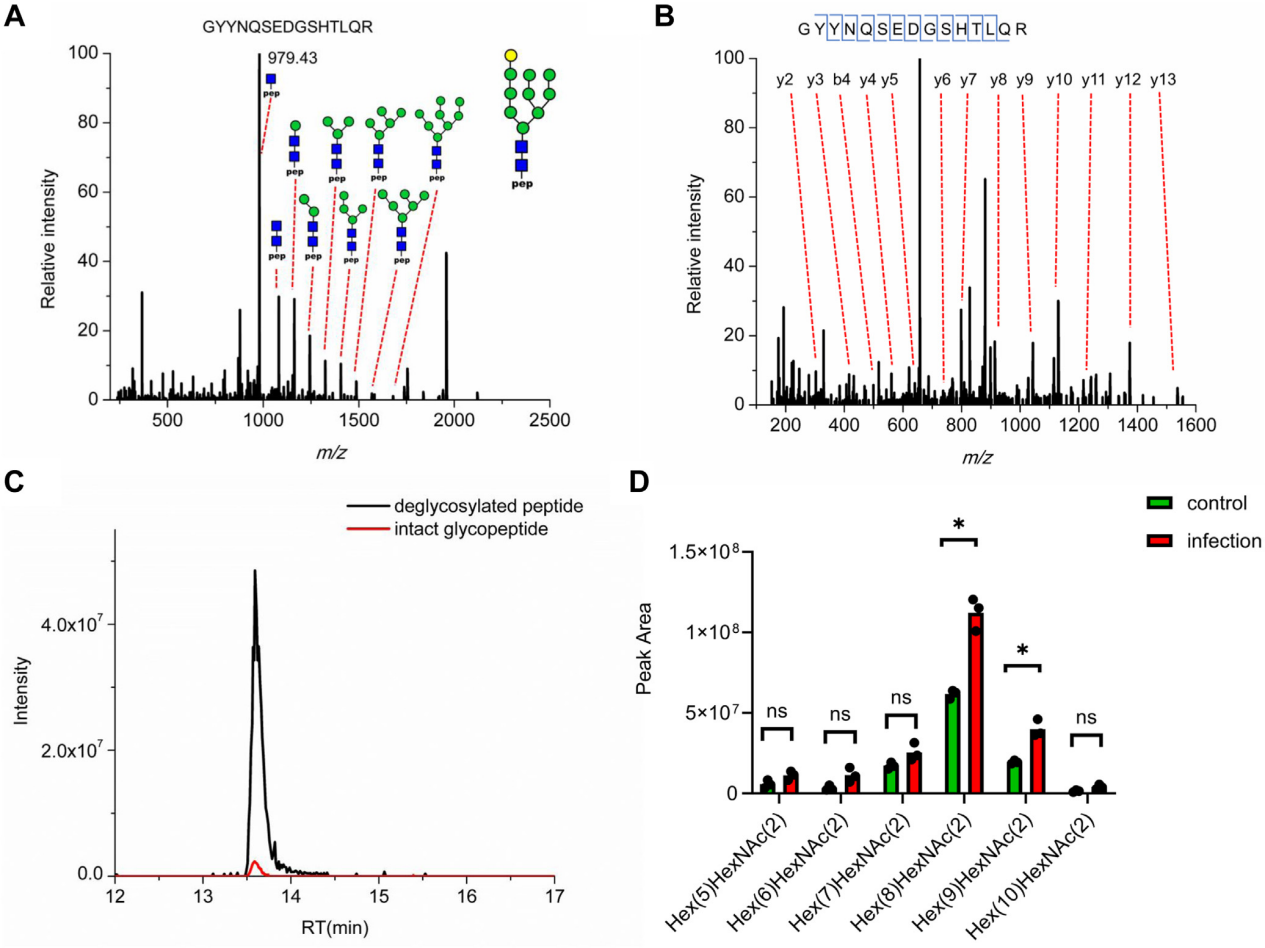
**Confirmation of C∗:07:02 allele with glycoproteomics analysis of HLA.***A*, annotated MS/MS spectra of intact glycopeptide from C∗:07:02 allele. *B*, MS/MS spectrum and sequence coverage of deglycosylated peptide GYYN#QSEDGSHTLQR. *C*, consistency between retention times from intact and deglycosylated peptides of C∗:07:02 allele. *D*, abundance changes of C∗:07:02 glycopeptides with different glycan compositions from the flow-through of MHC immunoprecipitation. HLA, human leukocyte antigen; MHC, major histocompatibility complex; MS/MS, tandem mass spectrometry.

Through the glycosylation analysis of HLAs from both immunoprecipitation and flow-through, mono-glucosylated glycopeptide from HLA-C∗:07 allele was identified along with C∗:15 allele (Fig. 5*A*). This was contradictory to a previous study that considered Calu-3 having homozygous C∗:15 alleles (26). The existence of C∗:07 allele was also identified from deglycosylated peptide (Fig. 5*B*) with strong evidence from the MS/MS spectra to differentiate it from the deglycosylated peptide of C∗:15 allele (Supplemental Fig. S11). The retention times of intact glycopeptide and deglycosylated peptide of C∗:07 allele correlated well and both of them differed from the retention time of glycopeptide from C∗:15 allele, which further verified the peptide sequence of intact glycopeptides of C∗:07 allele (Fig. 5*C*). The identification of C∗:07 allele can be also verified by the proteomics analysis of HLAs from MHC immunoprecipitation as multiple unique peptides were identified with high quality MS/MS spectra (Supplemental Fig. S12). These results were consistent with recent studies that analyzed the allele-specific abundance change of HLAs from Calu-3 cells during SARS-CoV-2 infection by transcriptomics and HLA genotyping from multiple independent studies (47, 48). Interestingly, the abundance changes of C∗:07 glycopeptides showed different patterns compared with that from C∗:15 glycopeptides as there were no significant increase of either mono-glucosylated glycopeptide or glycopeptides with fewer than five mannoses (Fig. 5*D*).

**Fig. 6:**
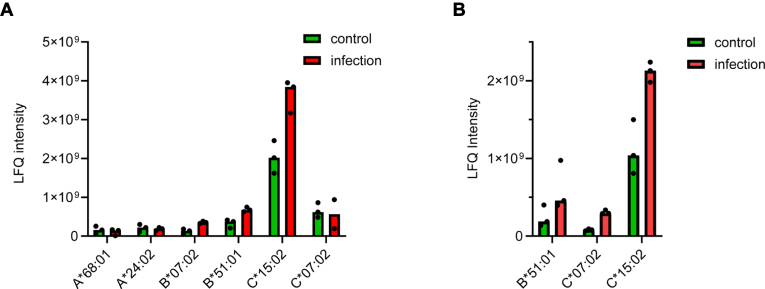
**Allele-specific abundance changes of HLA from the flow-through of MHC immunoprecipitation.***A*, intensities of the deglycosylated peptides from each allele identified by HILIC SPE based glycoproteomics approach. *B*, LFQ intensities of B∗51:01, C∗07:02, and C∗15:02 identified by total proteomics approach. HLA, human leukocyte antigen; HILIC SPE, hydrophilic interaction solid-phase extraction; LFQ, label-free quantification; MHC, major histocompatibility complex.

From the search results of proteomics analysis, many other HLA alleles were also identified through shared peptides with alleles from Calu-3 cells. The assignment of shared peptides to other alleles that do not exist in Calu-3 cells could interfere with the quantification of each allele by search engine. To reduce the ambiguity in proteomics identification of HLA alleles and improve the accuracy in relative quantification of each allele, we constructed a Calu-3-specific protein database by replacing the existing HLA-A, HLA-B, and HLA-C protein sequences from UniProt database with sequences of HLA alleles only from Calu-3 cells, including A∗68:01, A∗24:02, B∗07:02, B∗51:01, C∗07:02, and C15∗:02 that from IPD IMGT/HLA database (34). We firstly used this database to analyze the HLA proteins from the flow-through of immunoprecipitation. The N-glycoproteome data were searched against this customized database and all the N-glycosites from each allele were identified (Supplemental File S7). Relative quantification of each N-glycosite revealed the abundance change of HLA was allele-specific (Supplemental File S8). Significant higher level of B∗07:02, B∗51:01, and C∗15:02 was found from infected cells, while the level of both HLA-A alleles was very low from the flow-through of MHC immunoprecipitation (Fig. 6*A*). The results from N-glycoproteome were consistent with total proteome analysis, while HLA-A was not detected and higher level of B∗51:01, C∗07:02, and C∗15:02 was detected (Fig. 6*B*).

**Fig. 7:**
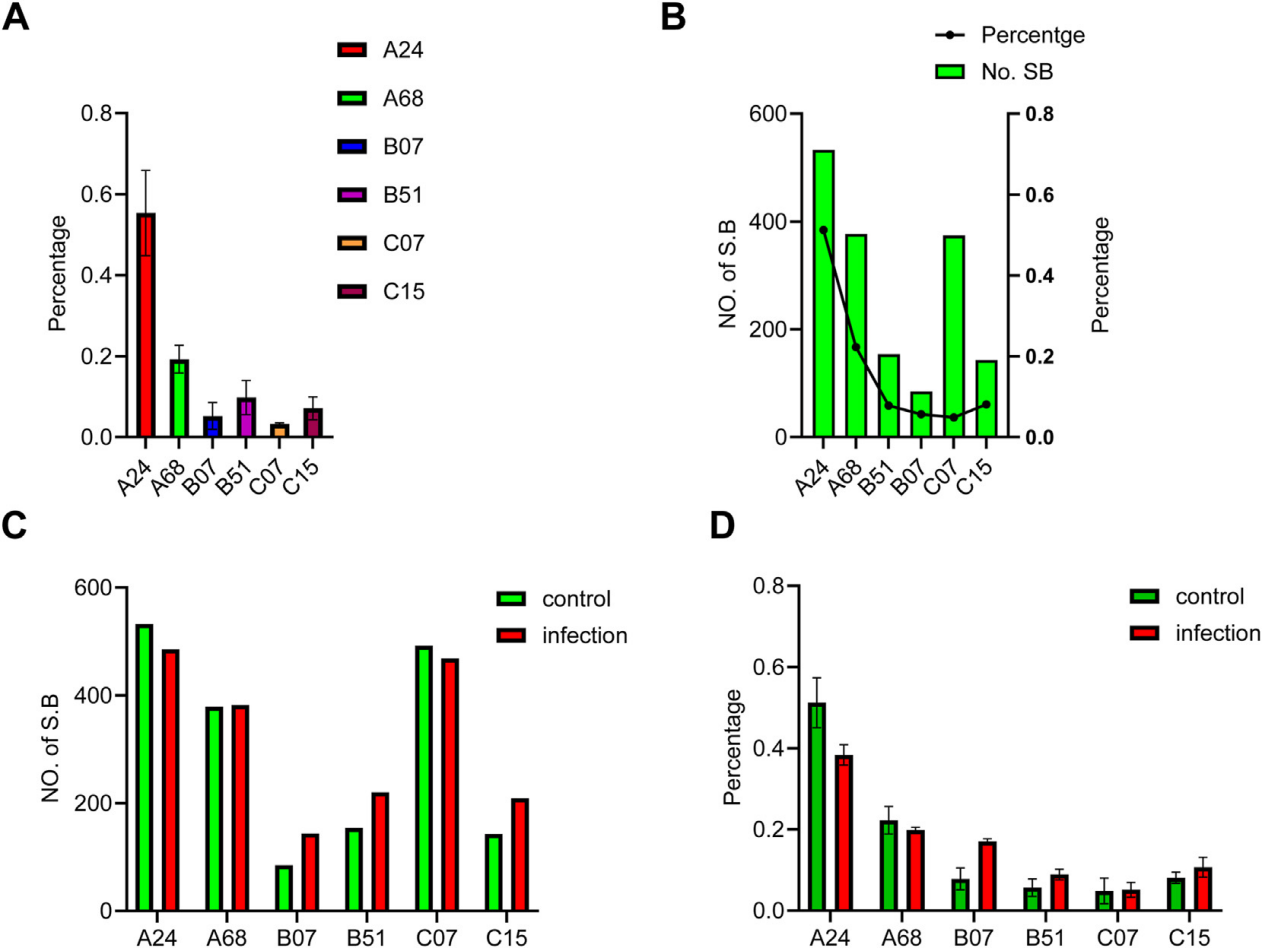
**Allele-specific abundance changes of HLA from the elution of MHC immunoprecipitation.***A*, relative quantification of each HLA allele by LFQ. *B*, correlation between allele abundance and antigen presentation. *C*, changes in the relative abundance of each allele correlates between control and infected cells. *D*, differences in antigen presentation of each allele between control and infected cells. An integrated immunopeptidomics and proteomics study of SARS-Cov-2 infection was established to comprehensively decipher the changes in host cells in response to viral infection. Immunopeptidomics analysis identified viral antigens presented by host cells through both class I and class II MHC system. Quantitative proteomics and glycoproteomics identified the activation of TLR3–interferon pathway. Glycosylation analysis of HLA proteins revealed dynamic changes in allele-specific abundance of HLA proteins that are associated with antigen presentation. HLA, human leukocyte antigen; LFQ, label-free quantification; MHC, major histocompatibility complex; SARS-CoV-2, severe acute respiratory syndrome coronavirus 2; TLR, toll-like receptor.

We also investigated any correlation between the abundance of each HLA allele and the antigens it presented using quantitative analysis of HLAs from immunoprecipitation. To normalize the abundance of each allele and simplify the comparison between control and infected cells, we used the percentage of the razor LFQ intensity of each allele among the total razor LFQ intensity of all alleles. It was found that the relative abundance of each allele was consistent among three independent replicates of immunoprecipitation with control cells (Fig. 7*A*) and the level of their abundance correlated with the number of strong 9mer binders to each allele with the exception of C∗07:02 (Fig. 7*B*), which was predicted by NetMHCpan4.0. Comparing the changes in relative abundance of each allele from control and infected cells revealed that there was an increased percentage of B∗51:01, B∗07:02, and C∗15:02 alleles (Fig. 7*C*). Interestingly, the changes in the percentage of each allele correlated well with the changes in the number of strong binders (Fig. 7*D*). These findings further demonstrated that the abundance changes of HLAs during SARS-CoV-2 infection in Calu-3 cells were allele-specific and impacted antigen presentation.

## Discussion

Host cell infected by coronavirus is the simplest model to study the mechanism of viral infection and the impact on the immune system for the development of vaccines and therapeutics. Although several immunopeptidomics and proteomics studies of SARS-CoV-2–infected cells have been reported (23, 24, 25, 26, 49), there is not yet a study that integrated both immunopeptidomics and proteomics, including PTM-targeted subomics. Thus, we developed a workflow that is comprised of immunopeptidomics, proteomics, glycoproteomics, and HLA-targeted glycosylation analysis to characterize how host cells response to infection that may attribute to the pathology of COVID-19.

With immunopeptidomics, one epitope from spike protein was identified from MHC I immunopeptidome, while epitopes from nucleocapsid protein were identified from both MHC I and MHC II. A few viral epitopes were also reported from previous study with Calu-3 cell lines and matched its HLA alleles (26). Additionally, this study provided the first identification of MHC II epitopes from nucleoprotein, and one of the peptides (SPDDQIGYYRRATRRIR) has a few overlaps with a class I epitope that has been identified from a previous study (NSSPDDQIGYY) and this epitope was found to be immunogenic in eliciting CD^8+^ T cells (13). Further studies are needed to investigate the immunogenicity of those viral epitopes from nucleocapsid protein. This protein could also be considered as a target for vaccine development since its sequence has been stable in the different variants of SARS-CoV-2. Fewer viral epitopes were identified from Calu-3 cells compared with a study using HEK293 and A549 cell lines that transfected with ACE2 and TMSSP2(19). We speculate that this could result from differences in HLA alleles among the cell lines as downregulation of surface MHC I was found in Calu-3 cells. To investigate how the antigen presentation was affected by viral infections, we performed total proteome analysis using the flow-through lysate from MHC immunoprecipitation. These experiments showed a higher level of HLA-B and HLA-C were found from infected cells, while the level of HLA-DR remained the same. The result seems contradictory to recent discoveries that showed MHC-I to be downregulated in infected cells and COVID-19 patients (28, 29). However, since the proteomics samples were from the flow-through of MHC immunoprecipitation, it is anticipated that the HLA proteins identified by proteomics analysis were actually free of MHC, and the increased abundance of HLA-B and HLA-C more likely resulted from disrupted MHC. This observation was consistent with the finding that MHC-Ι molecules were selectively targeted for lysosomal degradation *via* autophagy by ORF8, which was found in infected cells (28). In addition, we performed global and targeted glycosylation analysis of HLAs from both elution and flow-through of MHC immunoprecipitation. It was revealed that HLA proteins had different glycosylation patterns when associated with antigens on the cell surface compared when it was antigen-free. Higher level of mono-glucosylated HLA glycopeptide suggested that certain amount of HLA proteins was hindered from proper folding, resulting in the gradation of HLA protein and generation of trimmed glycopeptides with less than five mannoses. This finding was confirmed to some extent by a recent study in which ORF7 interacted with HLA and the level of Endo H–sensitive HLA increased in ORF7 overexpressed A549 cells (50). Higher level of improperly glycosylated HLAs in infected cells suggested that SARS-CoV-2 infection changed the folding and degradation of HLA proteins. However, this change was too subtle to be detected by either total proteomics or immunopeptidomics alone as suggested from previous studies. Interactomic studies of viral proteins showed that several viral proteins interact with HLAs in human cell lines (21, 51), however, those proteins were not identified in the digest of HLA proteins from MHC immunoprecipitation, suggesting they only bound to HLA proteins intracellularly.

Our findings confirmed that it is important to include multiple level of omics and analyze the data systematically. With the addition of quantitative glycoproteomics, we were able to discover that the level of ACE2 was decreased in infected cells, which was consistent with findings in animal models. However, ACE2 cannot be quantified in previous studies with cell lines by total proteomics (23). We discovered that strong innate immune response in infected cells could be initiated by TLR3, which senses viral RNA and triggers the activation of IFN signaling pathway. The increased expression of TLR3 suggested that the activation of IFN pathway was triggered by recognition of dsRNA from SARS-CoV-2, followed by secretion of cytokines. A recent study suggested the stimulation of INF pathway in SARS-CoV-2–infected Calu-3 cells was due to the lower expression of M protein. However, M protein also had a high expression in Calu-3 cells after 24 h. We hypothesized that activation of IFN pathway could be attributed to the higher expression of TLR3 in Calu-3 cells, which has higher transcription of TLR3 gene than Caco-2 and A549 cell lines in other proteomics studies (52). This assumption was partly supported by the finding that inhibition of histamine-induced expression of TLR3 in SARS-CoV-2–infected cells can reduce TLR3-dependent signaling processes and secretion of cytokines (53). This was the first study that links TLR3 with SARS-CoV-2 sensing specifically among other TLRs and TLR3 is well known to activate the production of inflammatory cytokines and trigger expression of IFN-inducible genes through the Janus kinase–STAT pathway (54). Among the three STAT members identified, STAT1 showed highest fold change and it was consistent with previous finding that STAT1 contributes to innate immune response during viral infection (55). Proteomics identification also suggested potential roles of ISGs in antiviral activity, including ISG20, which has exonuclease activity with specificity for ssRNA. The antiviral activity of IFN against RNA virus is partly mediated by ISG20 (56). It also discriminates between self and nonself-translation (57). Thus, we can propose that SARS-CoV-2 infection triggered the innate immune response in Calu-3 cells through TLR3–STAT1–INF–ISG axis.

The result from this integrated study might be able to explain the imbalanced innate and adaptive immune response of SARS-CoV-2 infection, which was not reported in other similar proteomics study with different cell lines. However, the imbalanced allele–specific transcription of HLA was suggested from transcriptomics study, especially HLA-B and HLA-C alleles in infected Calu-3 cells (47, 48). The expression difference of different alleles was confirmed, and changes in antigen presentation was also revealed in this study by MS. Thus, we suspect that the impact of viral infection on antigen presentation could be HLA allele dependent as the association between HLA allele frequencies and susceptibility to COVID19 in large population was suggested by a few studies (58, 59). Interestingly, one HLA allele of Calu-3 cells, HLA-B∗51:01, has been found in association with severe disease and worse outcomes in Chinese population with COVID-19 (60). The impaired antigen presentation with these HLA alleles may lead to the exhaustion of CD^8+^ T cells in patients with severe symptoms (30), while CD^4+^ T cell response can be found in most patients and unexposed individuals (8). This was consistent with our finding that the presentation of MHC II antigens was not affected in infected cells. The mechanism of downregulation of MHC I antigen presentation was further studied by comprehensive analysis of HLA glycosylation as higher level of improper glycosylation of C∗15:02 allele was found in infected cells, which was also reported recently. Expanded analysis of proteomics and glycoproteomics data suggested that this impact could be allele-specific. A noticeable difference in the abundance change from each allele was observed, which affected the antigen presentation during SARS-CoV-2 infection. Expanded application of the established approach to cell lines with different HLA haplotypes and clinical samples with accurate HLA genotyping can shed more lights on how impaired antigen presentation contribute to the pathology of COVID-19. Overall, this study, combined with many others, will collectively help develop a precision strategy for vaccination and therapy.

## Data Availability

Proteomics data have been deposited to the ProteomeXchange Consortium *via* the PRIDE (61) partner repository. Immunopeptidomics and intact glycopeptide data can be found with the dataset identifier PXD036022 (Username: reviewer_pxd036022@ebi.ac.uk Password: IY1B31Fz). Total proteomics and N-glycoproteomics data can be found with dataset identifier PXD037097(Username: reviewer_pxd037097@ebi.ac.uk Password: U5riYH1t).

## Supplemental data

This article contains supplemental data.

## Conflict of interest

The authors declare no competing interests.
