# Supplementary material for: Integrated Immunopeptidomics and Proteomics Study of SARS-CoV-2–Infected Calu-3 Cells Reveals Dynamic Changes in Allele-specific HLA Abundance and Antigen Presentation
Source: Mol Cell Proteomics. 2023 Sep 13;22(10):100645. doi: 10.1016/j.mcpro.2023.100645 (PMC10580047; doi:10.1016/j.mcpro.2023.100645)
Supplement: Supplementary Figure [file mmc9.pptx]

## Slide 1
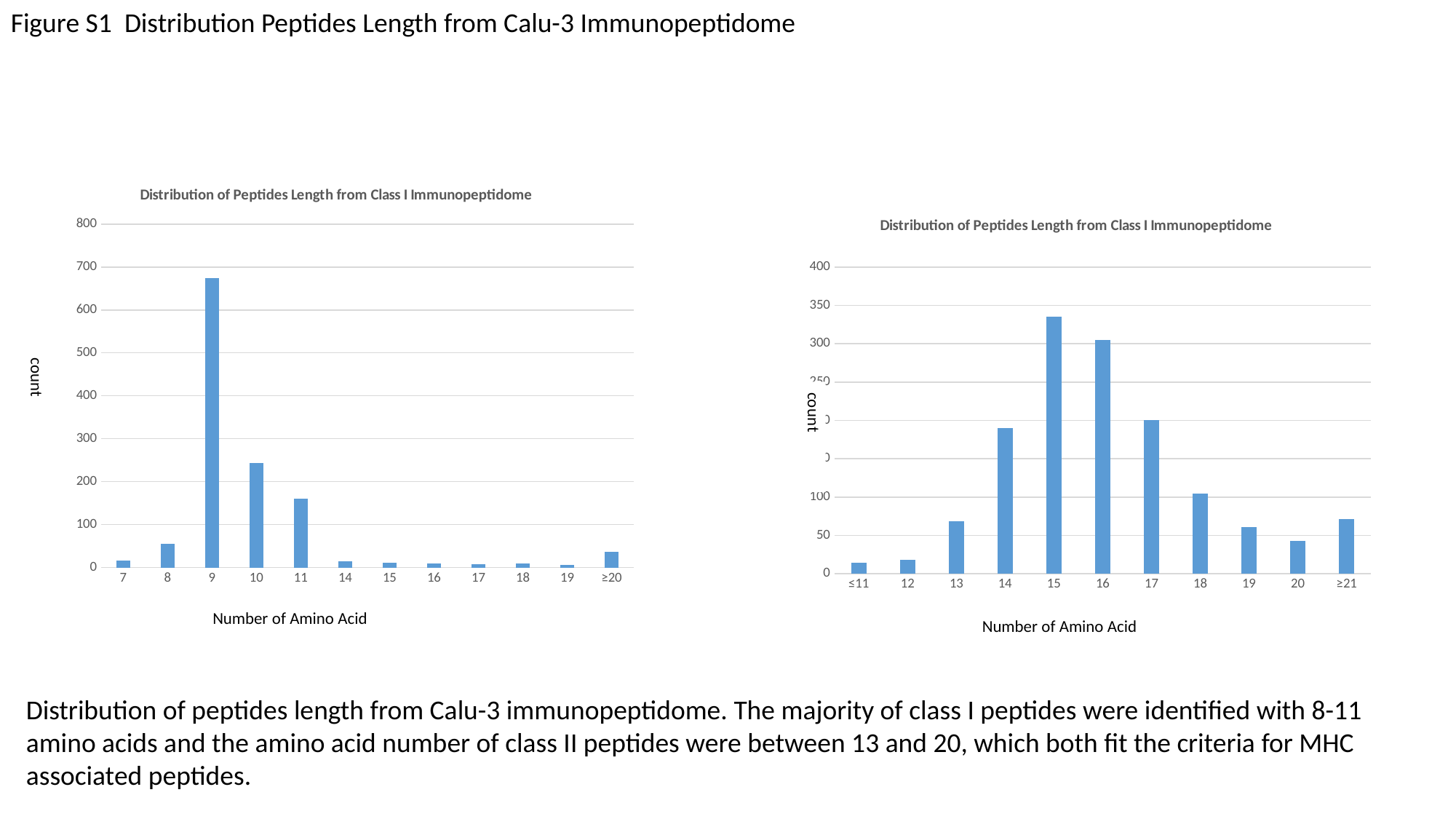

Figure S1 Distribution Peptides Length from Calu-3 Immunopeptidome
### Chart: Distribution of Peptides Length from Class I Immunopeptidome
| Category | Count |
|---|---|
| 7 | 17.0 |
| 8 | 56.0 |
| 9 | 675.0 |
| 10 | 244.0 |
| 11 | 160.0 |
| 14 | 14.0 |
| 15 | 11.0 |
| 16 | 9.0 |
| 17 | 7.0 |
| 18 | 9.0 |
| 19 | 6.0 |
| ≥20 | 37.0 |
### Chart: Distribution of Peptides Length from Class I Immunopeptidome
| Category | Count |
|---|---|
| ≤11 | 14.0 |
| 12 | 18.0 |
| 13 | 68.0 |
| 14 | 190.0 |
| 15 | 335.0 |
| 16 | 305.0 |
| 17 | 200.0 |
| 18 | 104.0 |
| 19 | 61.0 |
| 20 | 43.0 |
| ≥21 | 71.0 |count
Number of Amino Acid
Number of Amino Acid
Distribution of peptides length from Calu-3 immunopeptidome. The majority of class I peptides were identified with 8-11 amino acids and the amino acid number of class II peptides were between 13 and 20, which both fit the criteria for MHC associated peptides.

## Slide 2
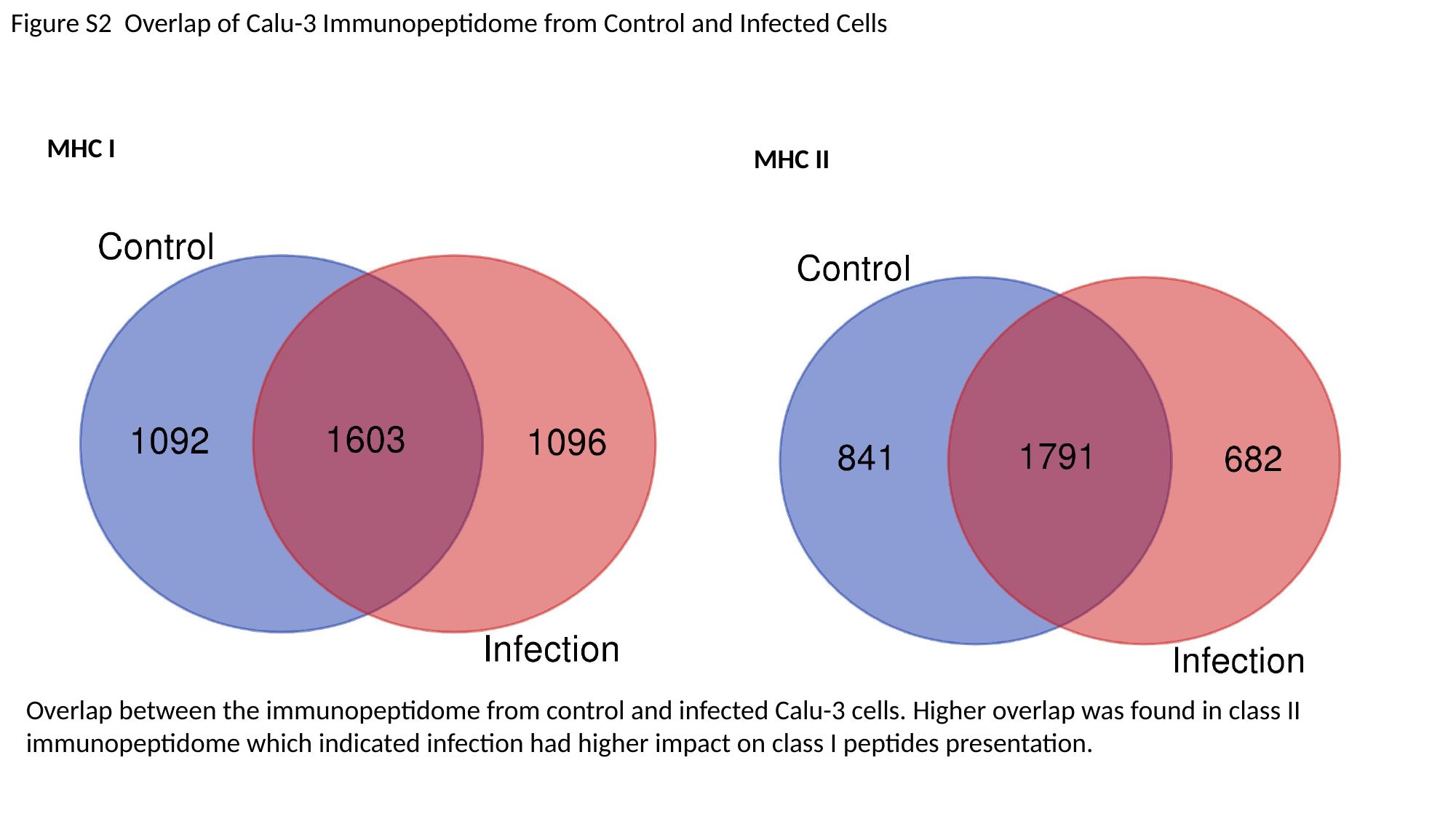

Figure S2 Overlap of Calu-3 Immunopeptidome from Control and Infected Cells
MHC I
MHC II
Overlap between the immunopeptidome from control and infected Calu-3 cells. Higher overlap was found in class II immunopeptidome which indicated infection had higher impact on class I peptides presentation.

## Slide 3
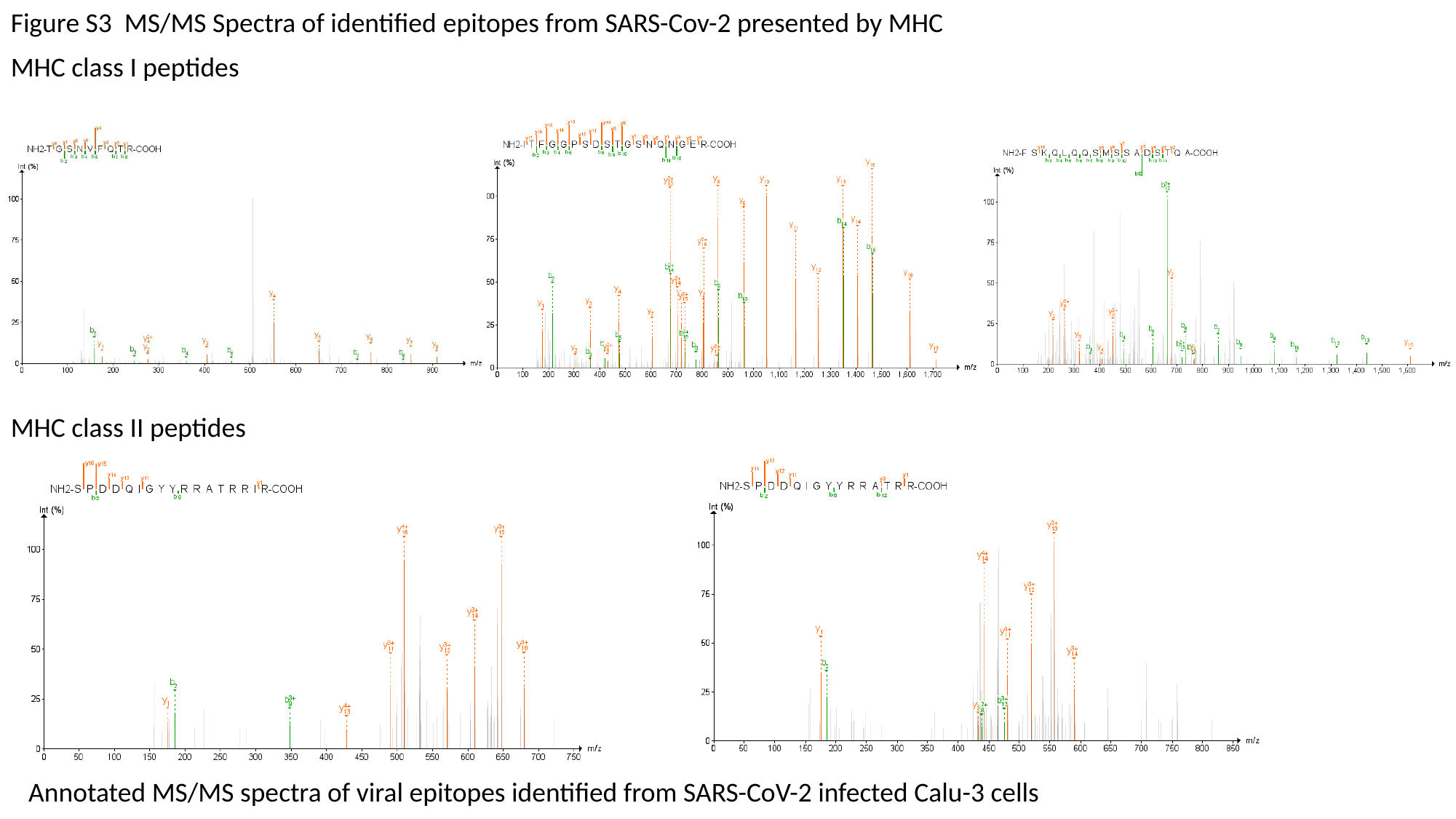

Figure S3 MS/MS Spectra of identified epitopes from SARS-Cov-2 presented by MHC
MHC class I peptides
MHC class II peptides
Annotated MS/MS spectra of viral epitopes identified from SARS-CoV-2 infected Calu-3 cells

## Slide 4
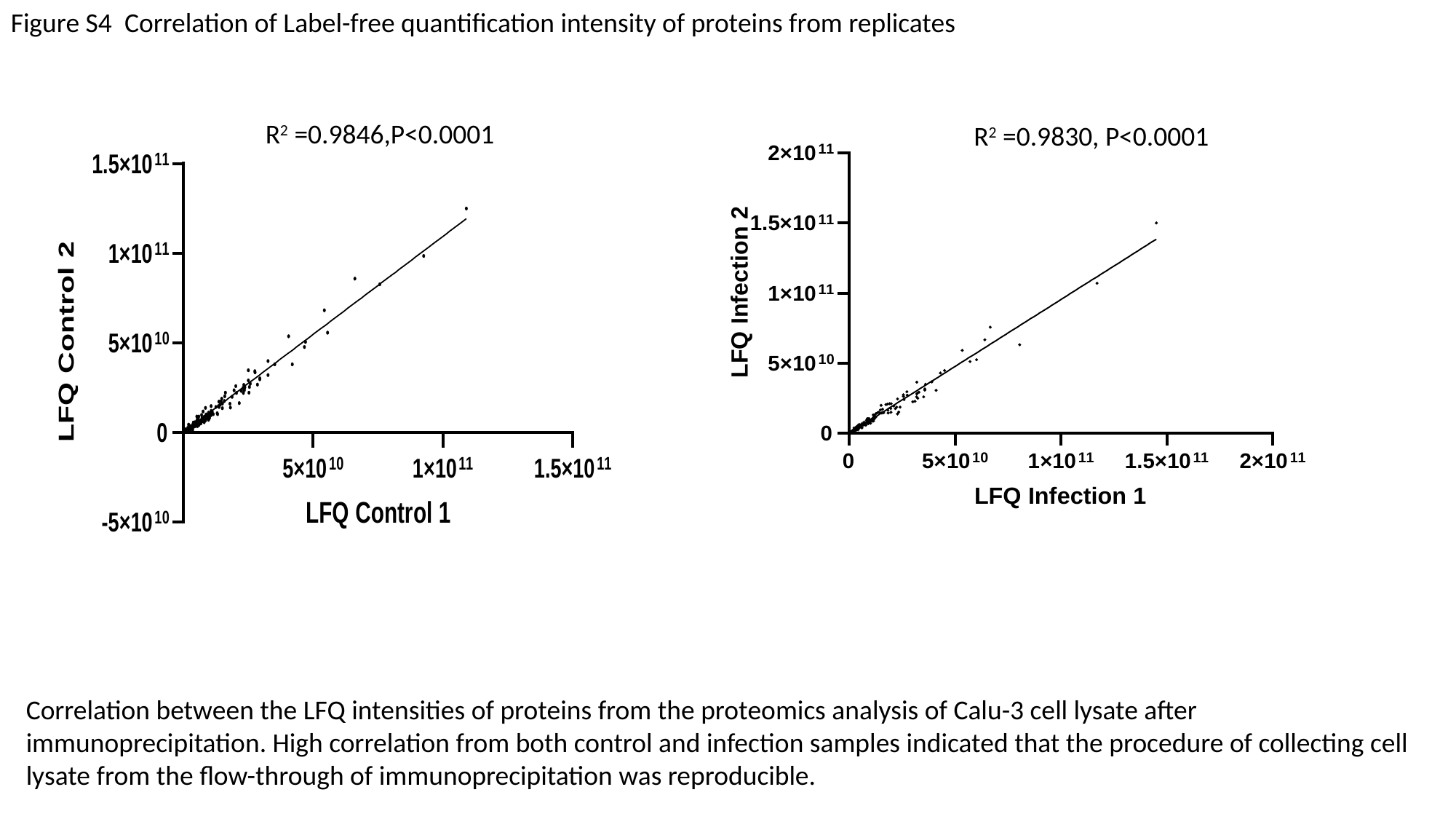

Figure S4 Correlation of Label-free quantification intensity of proteins from replicates
R2 =0.9846,P<0.0001
R2 =0.9830, P<0.0001
Correlation between the LFQ intensities of proteins from the proteomics analysis of Calu-3 cell lysate after immunoprecipitation. High correlation from both control and infection samples indicated that the procedure of collecting cell lysate from the flow-through of immunoprecipitation was reproducible.

## Slide 5
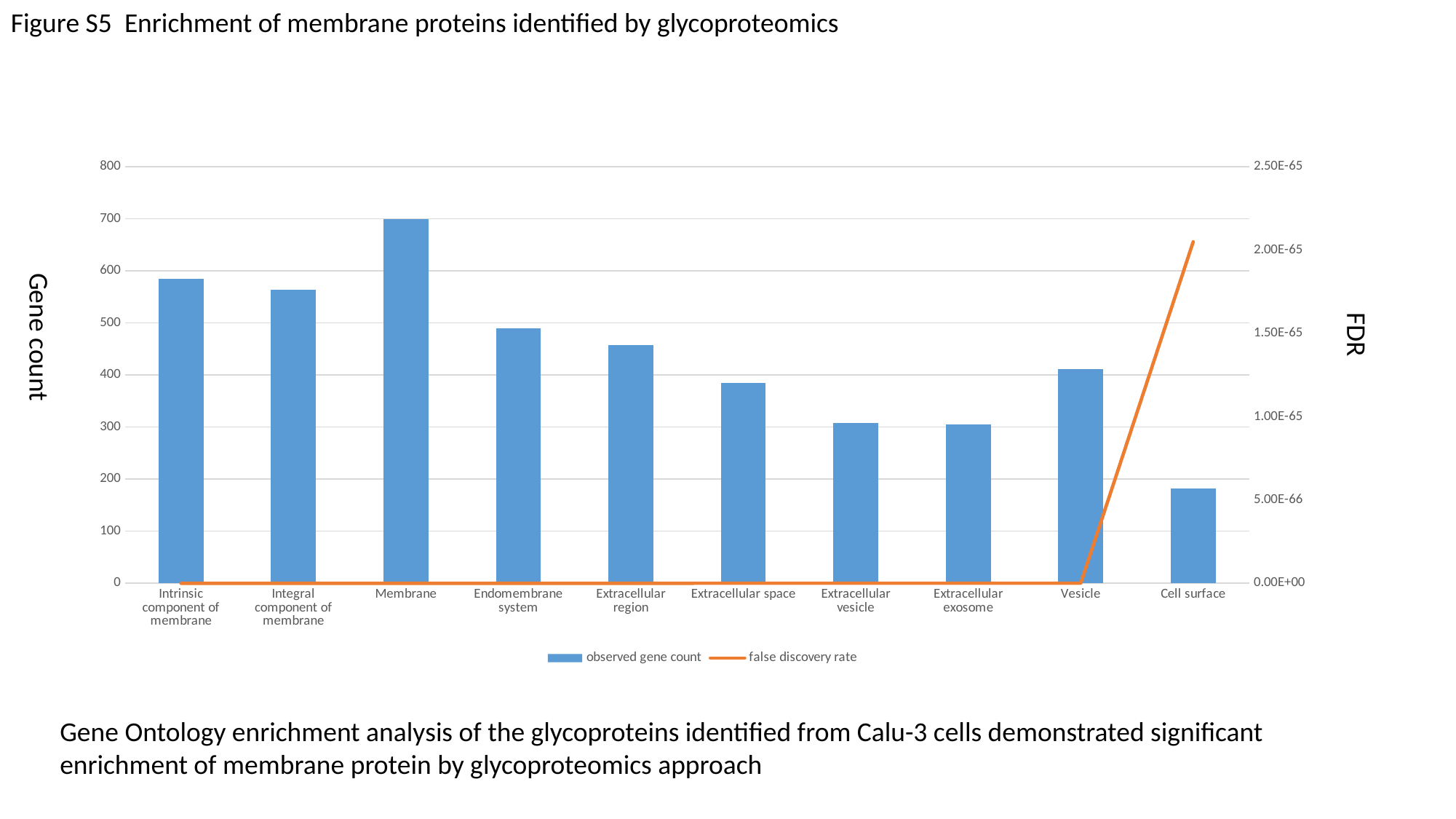

Figure S5 Enrichment of membrane proteins identified by glycoproteomics
### Chart
| Category | observed gene count | false discovery rate |
|---|---|---|
| Intrinsic component of membrane | 585.0 | 3.61e-129 |
| Integral component of membrane | 564.0 | 1.81e-120 |
| Membrane | 699.0 | 3.26e-96 |
| Endomembrane system | 490.0 | 1.28e-94 |
| Extracellular region | 458.0 | 4.06e-88 |
| Extracellular space | 385.0 | 1.67e-79 |
| Extracellular vesicle | 308.0 | 5.58e-77 |
| Extracellular exosome | 305.0 | 3.19e-76 |
| Vesicle | 412.0 | 1.58e-71 |
| Cell surface | 182.0 | 2.05e-65 |Gene count
FDR
Gene Ontology enrichment analysis of the glycoproteins identified from Calu-3 cells demonstrated significant enrichment of membrane protein by glycoproteomics approach

## Slide 6
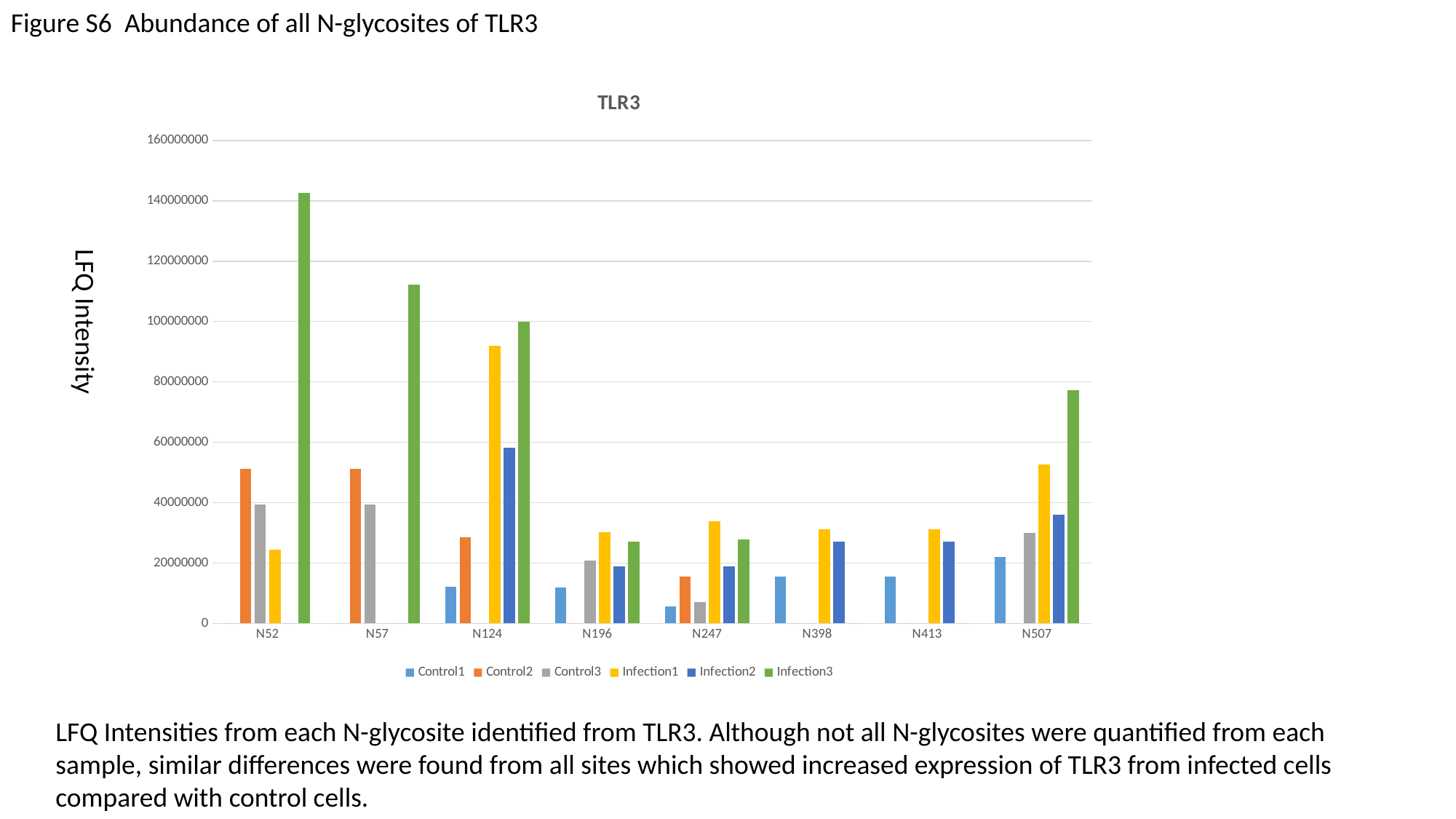

Figure S6 Abundance of all N-glycosites of TLR3
### Chart: TLR3
| Category | Control1 | Control2 | Control3 | Infection1 | Infection2 | Infection3 |
|---|---|---|---|---|---|---|
| N52 | 0.0 | 51201000.0 | 39290000.0 | 24508000.0 | 0.0 | 142650000.0 |
| N57 | 0.0 | 51201000.0 | 39290000.0 | 0.0 | 0.0 | 112160000.0 |
| N124 | 12248000.0 | 28444000.0 | 0.0 | 92080000.0 | 58225000.0 | 99939000.0 |
| N196 | 11839000.0 | 0.0 | 20820000.0 | 30107000.0 | 18933000.0 | 27086000.0 |
| N247 | 5678800.0 | 15387000.0 | 7058500.0 | 33807000.0 | 18978000.0 | 27752000.0 |
| N398 | 15624000.0 | 0.0 | 0.0 | 31145000.0 | 27148000.0 | 0.0 |
| N413 | 15624000.0 | 0.0 | 0.0 | 31145000.0 | 27148000.0 | 0.0 |
| N507 | 22070000.0 | 0.0 | 30025000.0 | 52758000.0 | 36113000.0 | 77258000.0 |LFQ Intensity
LFQ Intensities from each N-glycosite identified from TLR3. Although not all N-glycosites were quantified from each sample, similar differences were found from all sites which showed increased expression of TLR3 from infected cells compared with control cells.

## Slide 7
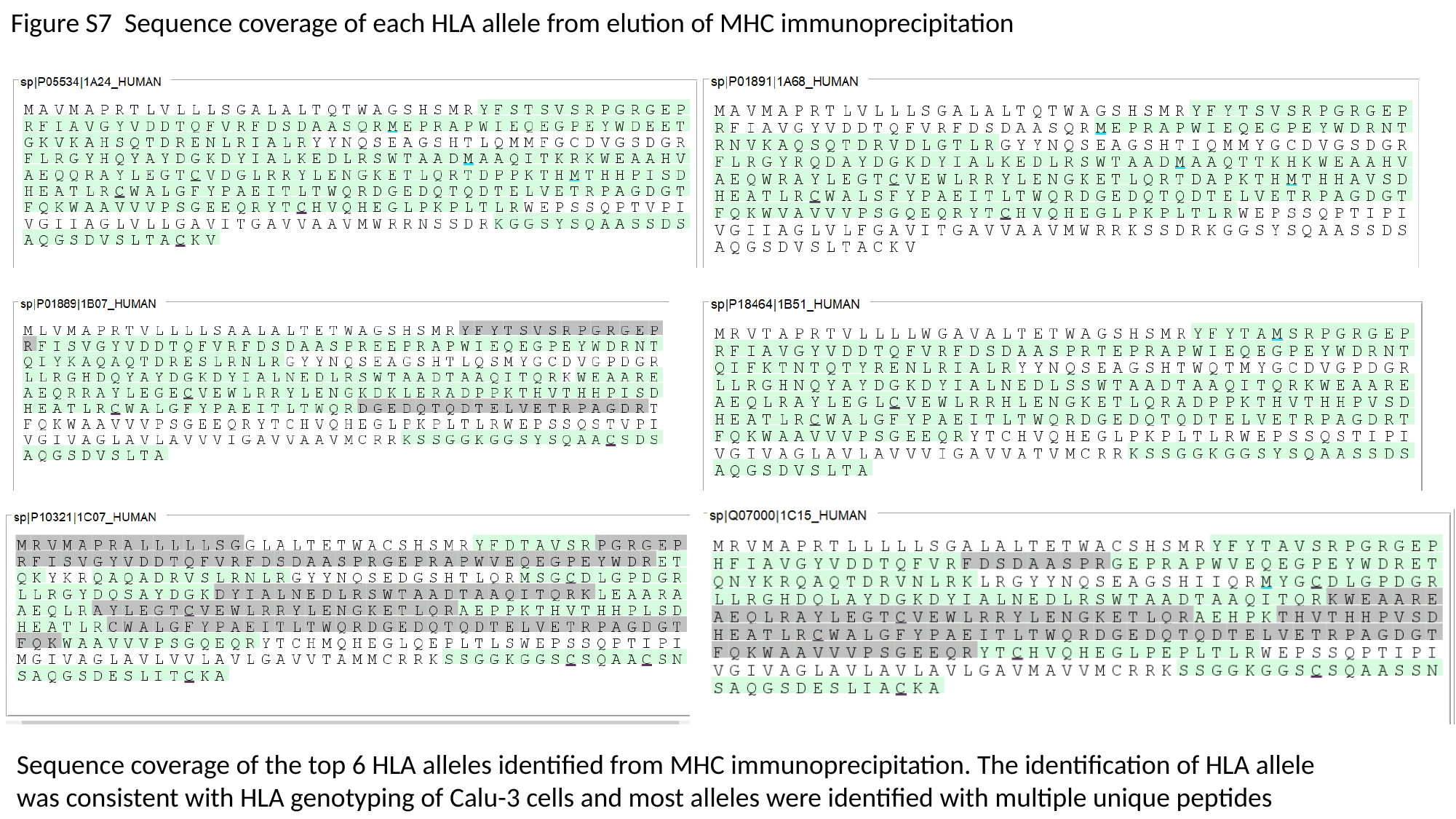

Figure S7 Sequence coverage of each HLA allele from elution of MHC immunoprecipitation
Sequence coverage of the top 6 HLA alleles identified from MHC immunoprecipitation. The identification of HLA allele was consistent with HLA genotyping of Calu-3 cells and most alleles were identified with multiple unique peptides

## Slide 8
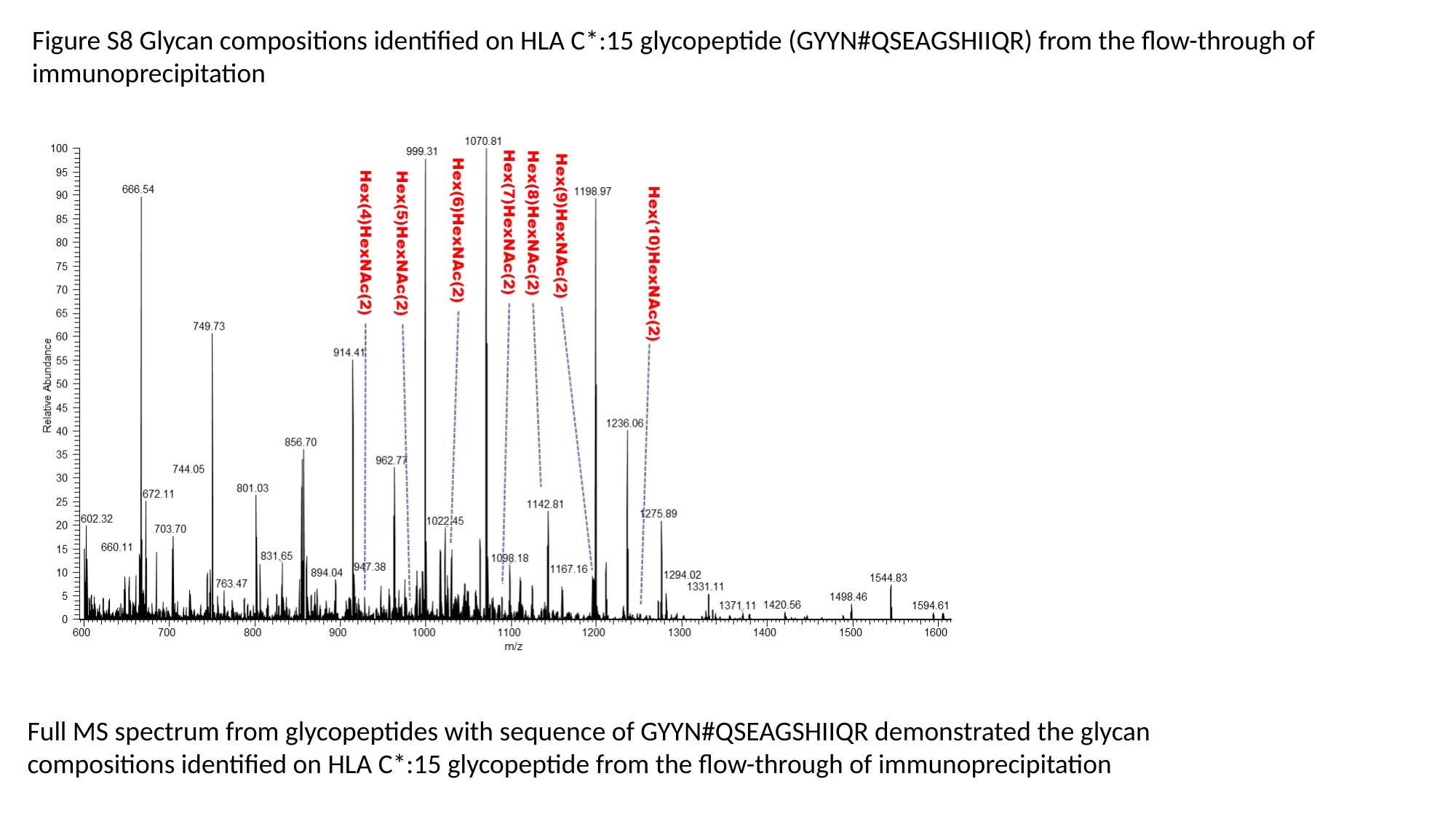

Figure S8 Glycan compositions identified on HLA C*:15 glycopeptide (GYYN#QSEAGSHIIQR) from the flow-through of immunoprecipitation
Full MS spectrum from glycopeptides with sequence of GYYN#QSEAGSHIIQR demonstrated the glycan compositions identified on HLA C*:15 glycopeptide from the flow-through of immunoprecipitation

## Slide 9
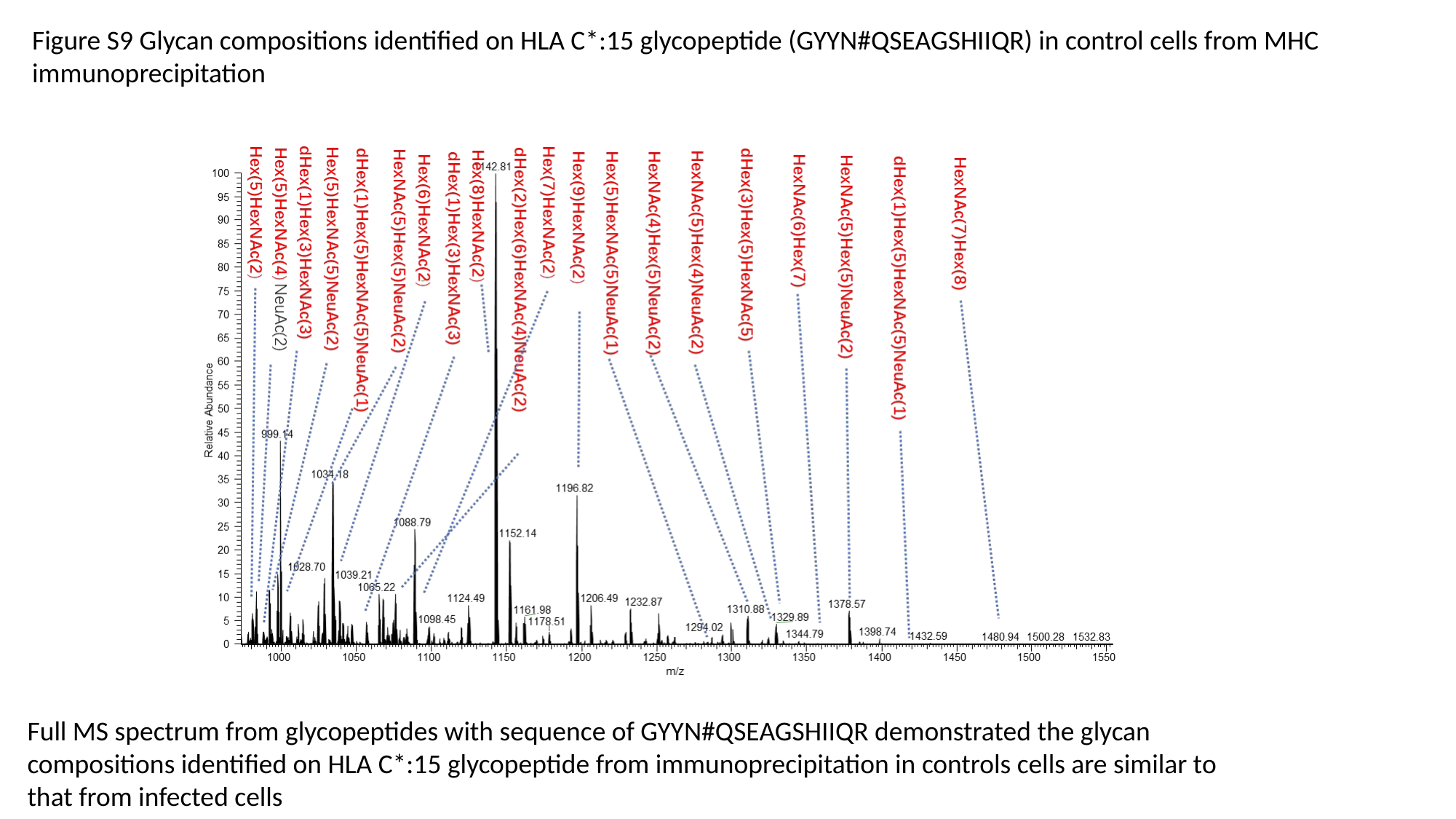

Figure S9 Glycan compositions identified on HLA C*:15 glycopeptide (GYYN#QSEAGSHIIQR) in control cells from MHC immunoprecipitation
Full MS spectrum from glycopeptides with sequence of GYYN#QSEAGSHIIQR demonstrated the glycan compositions identified on HLA C*:15 glycopeptide from immunoprecipitation in controls cells are similar to that from infected cells

## Slide 10
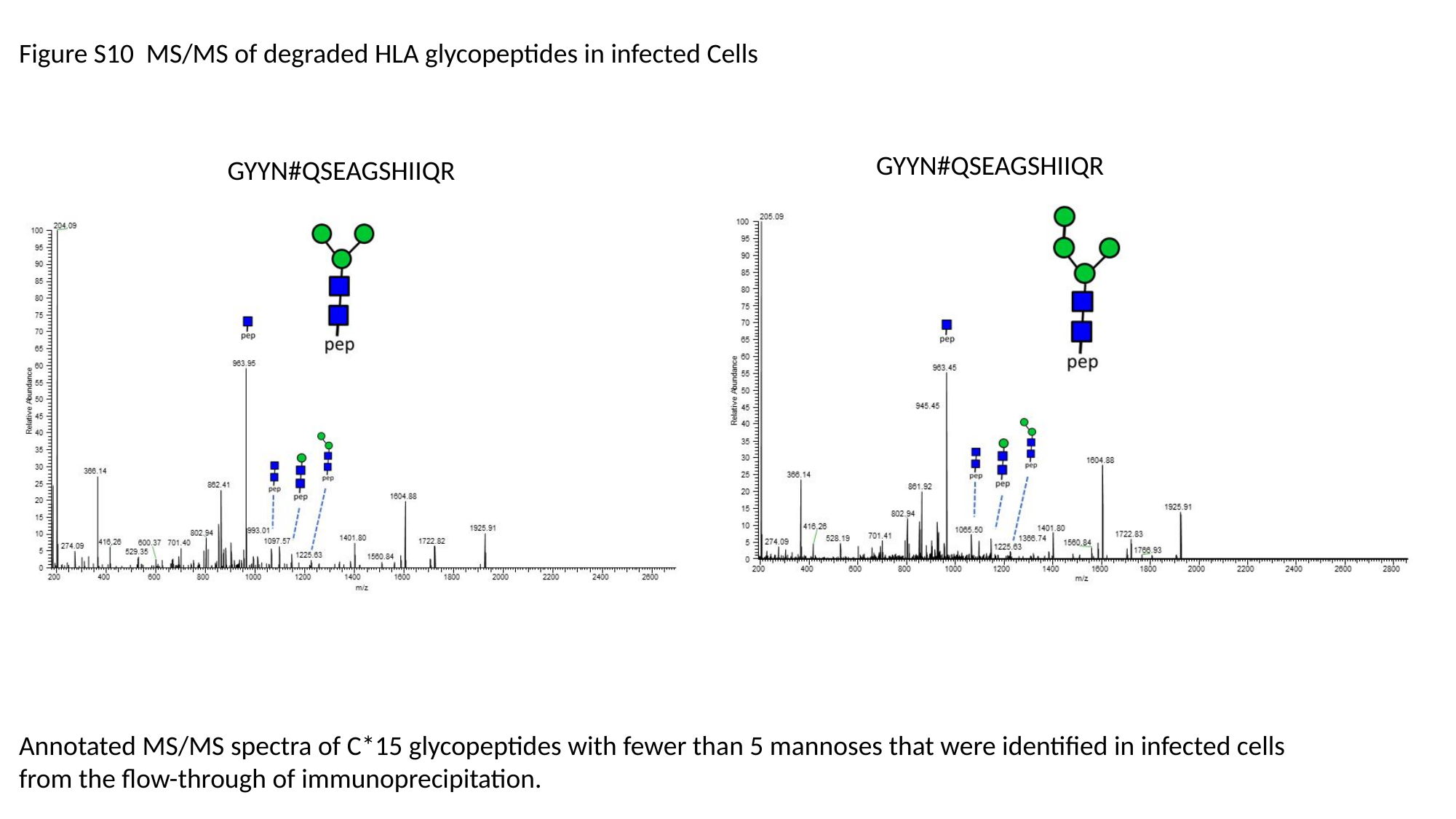

Figure S10 MS/MS of degraded HLA glycopeptides in infected Cells
GYYN#QSEAGSHIIQR
GYYN#QSEAGSHIIQR
Annotated MS/MS spectra of C*15 glycopeptides with fewer than 5 mannoses that were identified in infected cells from the flow-through of immunoprecipitation.

## Slide 11
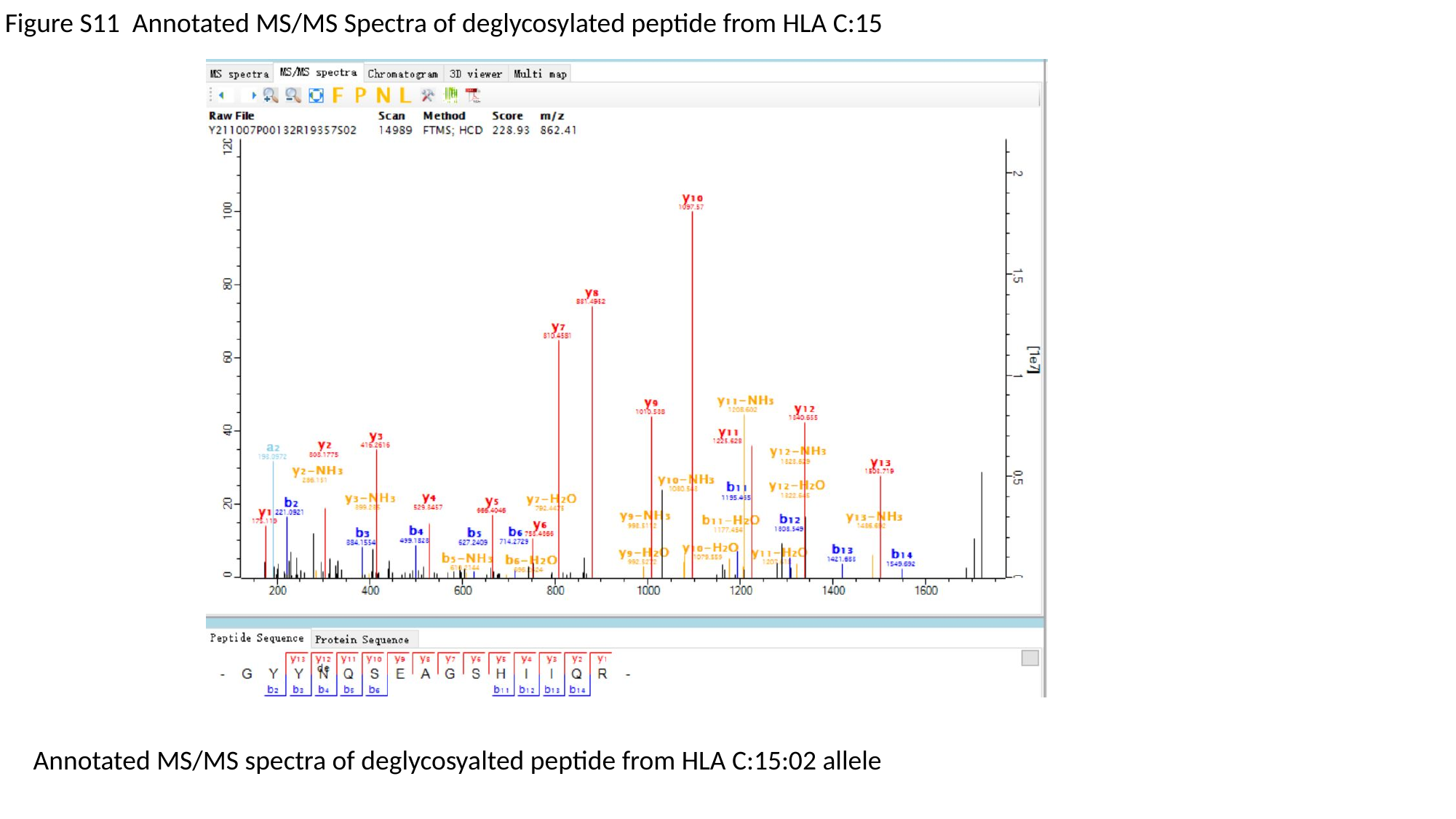

Figure S11 Annotated MS/MS Spectra of deglycosylated peptide from HLA C:15
Annotated MS/MS spectra of deglycosyalted peptide from HLA C:15:02 allele

## Slide 12
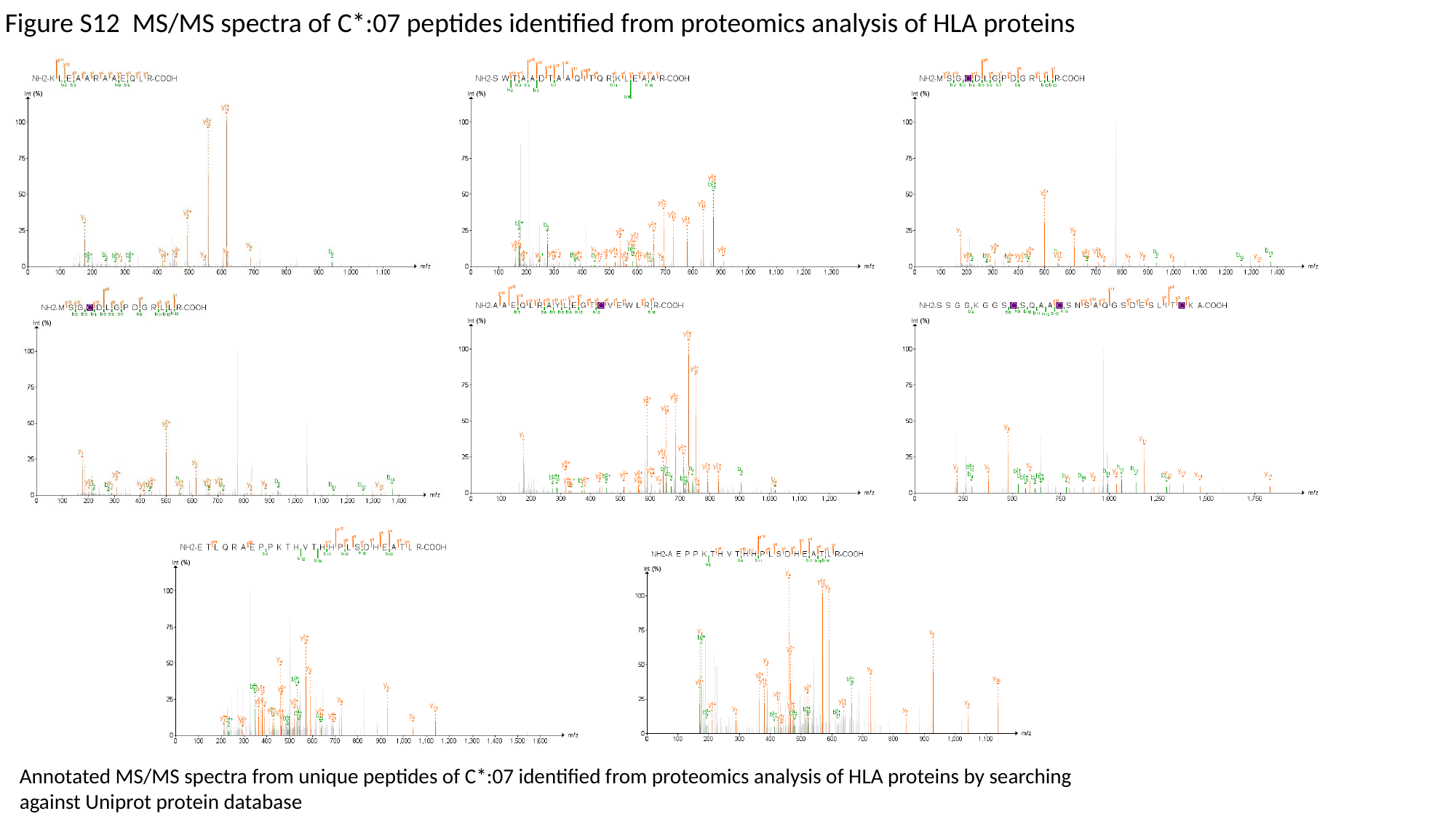

Figure S12 MS/MS spectra of C*:07 peptides identified from proteomics analysis of HLA proteins
Annotated MS/MS spectra from unique peptides of C*:07 identified from proteomics analysis of HLA proteins by searching against Uniprot protein database
